# All HER2-negative breast cancer patients need gBRCA testing: cost-effectiveness and clinical benefits

**DOI:** 10.1038/s41416-022-02111-y

**Published:** 2022-12-23

**Authors:** Huai-liang Wu, Zi-yin Luo, Zong-lin He, Yue Gong, Miao Mo, Wai-kit Ming, Guang-yu Liu

**Affiliations:** 1 Department of Breast Surgery, Key Laboratory of Breast Cancer in Shanghai, Department of Oncology, Fudan University Shanghai Cancer Center, Shanghai, China; 2 Department of Otorhinolaryngology Head and Neck Surgery, The Sixth Affiliated Hospital of Sun Yat‑sen University, Guangzhou, China; 3 Division of Life Science, The Hong Kong University of Science and Technology, Hong Kong SAR, China; 4 Department of Cancer Prevention, Fudan University Shanghai Cancer Center; Department of Oncology, Shanghai Medical College, Fudan University, Shanghai, China; 5 Department of Infectious Diseases and Public Health, Jockey Club College of Veterinary Medicine and Life Science, City University of Hong Kong, Hong Kong SAR, China

**Keywords:** Health care economics, Breast cancer

## Abstract

**Background:**

The OlympiA trial demonstrated the benefits of adjuvant usage of olaparib for high-risk patients with human epidermal growth factor receptor 2 (HER2)-negative breast cancer (BC) and germline BRCA (gBRCA) mutation. This provoked thoughts on the clinical criteria of gBRCA testing. This study aims to estimate the costs and benefits of gBRCA testing and adjuvant olaparib therapy for patients with triple-negative breast cancer (TNBC) and hormone-receptor (HR)-positive and HER2-negative BC in China and the United States of America (USA).

**Methods:**

We used a Markov chain decision tree analytic model to compare three gBRCA screening policies in China and the USA: (1) no gBRCA testing; (2) selected gBRCA testing and (3) universal gBRCA testing for nonmetastatic TNBC and HR-positive HER2-negative BC patients. We modelled the benefit of systemic therapy and risk-reducing surgeries among patients identified with pathogenic or likely pathogenic variants (PVs) in BRCA1 and BRCA2.

**Results:**

Changing from the selected gBRCA testing to the universal gBRCA testing in TNBC patients is cost-effective, with the incremental cost-effectiveness ratios (ICERs) being 10991.1 and 56518.2 USD/QALY in China and the USA, respectively. Expanding universal gBRCA testing to HR-positive HER2-negative BC and TNBC patients has ICERs of 2023.3 and 16611.1 USD/QALY in China and the USA, respectively.

**Discussion:**

By performing gBRCA testing on all HER2-negative BC patients, adjuvant olaparib can be offered to high-risk patients with a PV in BRCA1 or BRCA2. These patients are also candidates for risk-reducing surgeries, an important aspect of their survivorship care, and these interventions can improve survival outcomes. With the willingness-to-pay thresholds being 31,500.0 and 100,000.0 USD per QALY gained in China and the USA, respectively, universal gBRCA testing is likely cost-effective for all HER2-negative BC patients. This simplified criterion of gBRCA testing for BC is recommended for adoption by current guidelines in China and the USA.

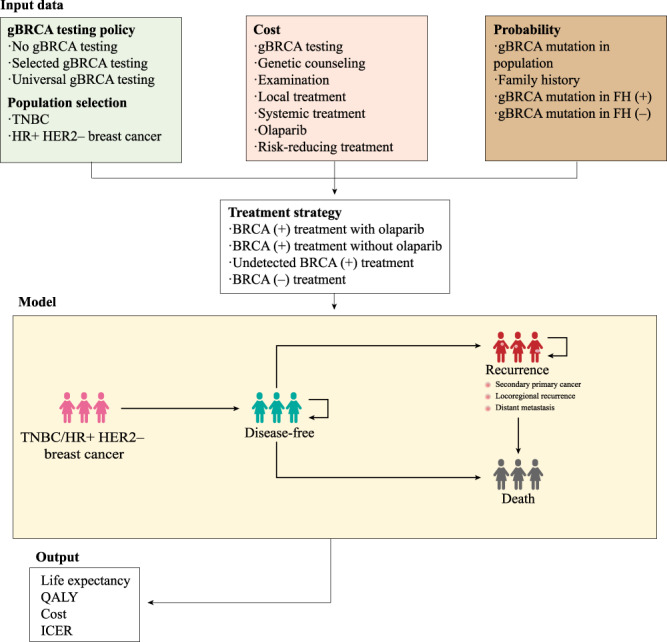

## Introduction

Germline BRCA (gBRCA) 1/2 pathogenic or likely pathogenic variants (P/PVs) tend to cause homologous recombination repair (HRR) deficiency in breast cancer (BC) patients, which may significantly expedite disease progression, leading to primary ovarian cancers, secondary contralateral BCs, locoregional recurrence and even distant metastasis [1, 2]. Approximately 10% of all triple-negative breast cancer (TNBC) patients carry gBRCA PVs [3–5], while ~9% belong to human epidermal growth factor receptor 2 (HER2)-negative BC patients [5]. Despite the similar survival outcomes observed in BRCA mutation carriers and noncarriers, risk-reducing operations (RROs) could provide a survival advantage for carriers with gBRCA PVs [6].

Poly-(ADP)-ribose polymerase (PARP) inhibition is synthetically lethal with a deficiency in HRR; therefore, PARP inhibitors (PARPi) are regarded as a promising approach for treating BRCA1/2-mutated BCs [7]. The clinical use of PARPi in BC was first reported in 2009 by the OlympiAD study, and olaparib was the first to receive the United States of America (USA) Food and Drug Administration (FDA) approval for treating metastatic breast cancer (MBC) [8]. Furthermore, a recent clinical trial, OlympiA, corroborated the benefits of PARPi in the adjuvant setting for high-risk HER2-negative BC patients with gBRCA PVs, calling for revisiting the criteria for the gBRCA testing [9, 10]. Due to the OlympiA study, olaparib has recently been approved by the USA FDA for the adjuvant treatment of high-risk early breast cancer [11].

To date, the American Society of Breast Surgeons recommends that germline BRCA testing is indicated for all newly diagnosed breast cancer patients [12]. The National Comprehensive Cancer Network (NCCN) and the American Society of Clinical Oncology (ASCO) recommend that any breast cancer patients younger than the age of 50 or those with a family history of breast and ovarian cancer or other high-risk factors undergo gBRCA screening testing [13, 14]. Moreover, following the OlympiaA study, the St. Gallen International Consensus Panel recommends genetic testing of patients meeting the OlympiA trial criteria, which are patients with stage I-IV TNBC and all patients with HR-positive HER2-negative stage II–IV breast cancer [15]. In China, the expert consensus of gBRCA testing recommends that patients younger than the age of 45 and those with high-risk family histories undergo gBRCA screening testing [16]. Genetic counselling is recommended before the patients undergo the gBRCA screening testing [17].

Due to the decreased cost of gBRCA testing and the promising efficacy of olaparib therapy to improve survival in patients with breast cancer and BRCA1/2 PVs, it is essential to examine whether gBRCA testing is appropriate for all patients with TNBC and HR-positive HER2-negative breast tumours [9, 10, 18]. Experts in the field have advocated for increasing access to gBRCA testing worldwide [19]. Given the possible cost-effectiveness of genetic screening and family history-based genetic testing in breast cancer, the focus of our study is to investigate a comprehensive gBRCA testing strategy among patients with breast cancer using a practically simplified screening approach and adjuvant use of olaparib.

In this study, we designed a cost-effectiveness analysis based on clinical data and compared three gBRCA testing strategies in all TNBC and HR-positive HER2-negative BC patients: (1) no gBRCA testing (no test); (2) selected gBRCA testing based on family history and genetic consultations (selected test) and (3) universal gBRCA testing for all TNBC and HR-positive HER2-negative BC patients (universal test). The analysis was performed explicitly based on China and the USA. The present study aimed to address the cost-effectiveness of BRCA-associated treatment (adjuvant olaparib and RRO) secondary to gBRCA testing in TNBC and all HER2-negative BC (inclusive of TNBC) patients under different scenarios in China and the USA.

## Subjects and methods

### Model design

TreeAge Pro 2019 (TreeAge, Williamstown, Massachusetts) was used for the Markov model building. The parameters included in this analysis are presented in Table S1. The detailed methods for model building and parameter estimation are described in Annex I.

**Fig. 1:**
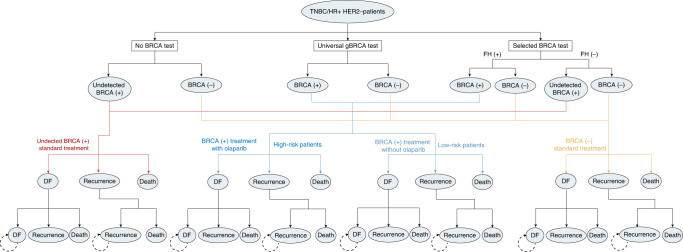
Model structure of cost-effectiveness analysis on different gBRCA testing policies. FH family history, DF disease-free state.

A decision tree analytic model based on a transitional Markov chain with clinical data from the Olympia trial and other studies was developed to estimate the costs and benefits of the germline BRCA mutation testing for a representative cohort of TNBC and HR-positive HER2-negative BC patients based mainly on the baseline demographic and clinical characteristics of the OlympiA trial [20]. The model consisted of three mutually exclusive health states, namely, disease-free (DF) (the early stage), recurrence (the advanced stage) [20] and death (Fig. 1 and Table S1). The DF state indicates the health state where a patient with invasive BC after resectable surgery does not experience events, the duration of which can be estimated by disease-free survival (DFS) [21]. The recurrence state represents the health state where the patient experiences locoregional recurrence or distant metastasis of the tumour from any cause. Death states result from the all-cause mortality of BC patients, so DF patients could also transition to death directly.

Each Markov model cycle was 1 year, and the time horizon was 20 years. Based on several epidemiological investigations of BC patients, the base-case model assumed that the mean age of the affected women is 40 years old [22, 23]. The model started when women diagnosed with TNBC or all HER2-negative BC encountered different scenarios of testing: universal testing, selected testing abiding by family history or no screening testing (Fig. 1). A positive gBRCA test plus high-risk stratification will prompt the delivery of olaparib as an adjuvant treatment.

To comply with the clinical situation, we assumed that the affected women would undergo standard treatment following the NCCN and the Chinese Anti-Cancer Association Committee of Breast Cancer Society (CACA-BC) guidelines. Standard treatment is defined as local treatment plus systemic treatment following the current guidelines for BC treatment after diagnosis. Three therapeutic strategies were considered throughout the model: (1) BRCA-negative patients would receive standard treatment; (2) undetected BRCA-positive patients would receive standard treatment with increased risks of bilateral breast cancer and endometrial cancer; (3) BRCA-positive patients would receive standard treatment, wherein high-risk patients would receive PARPi and RROs (for details, refer to Annex I).

Once recurrence is experienced in BRCA-mutant BCs, olaparib will be administered and maintained for 1 year [13]. However, if these BRCA-mutant BC have already received adjuvant olaparib, they would not be given again upon recurrence, considering the drug tolerance. The recurrence probability was calculated and merged from 3 possible recurrence states (locoregional recurrence, secondary primary malignancies and distant metastasis) reported in the literature (Tables S2, S3). All patients were expected to receive the best supportive treatment to curb recurrence until death. The annual mortality rate per cycle was calculated from the 5-year mortality rate reported by the OlympiA and relevant clinical trials [20], and all rates were converted into annual probabilities using the rate to probability formula (1 − e ^− rate × time^) where relevant.

The primary outcomes are quality-adjusted life-years (QALYs) gained and incremental cost-effectiveness ratios (ICERs) [24]. Secondary outcomes include life expectancy gained and survival outcomes. We applied half-cycle corrections to cost and effectiveness values in the model. The ICERs were compared between different strategies of the gBRCA testing based on willingness-to-pay (WTP). The WTP threshold was an estimate of how much governments, insurers and researchers were willing to pay for the health benefit, 31,500.0 USD/QALY for China and 100,000.0 USD/QALY for the USA, respectively [25, 26].

### Cost and utility estimates

This cost-effectiveness analysis was performed from a healthcare sector perspective, where only direct medical costs were considered in the model, including surgery, medicine, administration, lab tests, imaging and management of adverse event costs. The repertoire of costs is listed in Table S4, and details of the cost calculation are described in the Annex. The base costs were inflated to reflect 2021 USD, and the costs and utilities were discounted at a 3% annual rate to account for inflation [27].

The baseline utility values were adopted from previous reports of health utilities for BC patients [28, 29]. We set the base utility value for disease-free women without cancer at 0.85 [28]. We assumed the utility value to be 0.51 [30] for recurrence. We then modelled other utilities as a disutility from the baseline per previous literature [31–33]. After receiving olaparib, the utility of MBC recurrence was proposed to improve by 0.075, considering the prolonged progression-free survival in high-risk patients [8] (Supplementary Table S4).

### Sensitivity analysis

To address the uncertainty and evaluate the robustness of our results, one-way deterministic sensitivity analyses were performed. Each parameter was varied over the reported 95% confidence intervals (CIs), as shown in Table S1. In case of the lack of data for the 95% CI of a parameter, a variance of 10% of the variable itself from the base-case value for both the cost and utility would be assumed.

In addition, a probabilistic sensitivity analysis using a Monte Carlo simulation of 1000 trials from a prior defined probability distribution was conducted to assign the 95% CIs around the model outcomes to evaluate the stochastic effects of model input parameters [34]. In general, we assigned beta distributions to transition probabilities and utilities, gamma distributions to costs and log-normal distributions to relative risk. In the Monte Carlo simulation, we incorporated a wide range of utility inputs into the model (±10% of input values as SD for healthy and ±5% of input values as SD for recurrence).

Cost-effectiveness acceptability curves and scatter plots were developed from these simulations.

## Results

### Undergoing the universal gBRCA testing in patients with TNBC is cost-effective

**Fig. 2:**
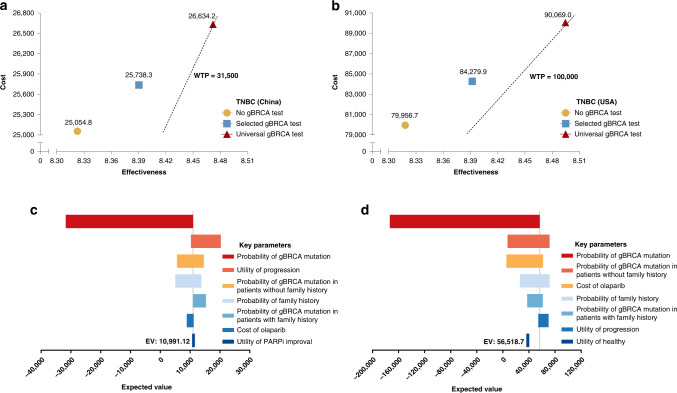
Cost-effectiveness analysis of different gBRCA testing policies in TNBC patients. **a** Cost-effectiveness ranking (China); **b** Cost-effectiveness ranking (USA); **c** Tornado diagram—net monetary benefits in the sensitivity analysis (China); **d** Tornado diagram—net monetary benefits in the sensitivity analysis (USA).

The model outputs were stable and robust to a wide range of model inputs and were estimated based on clinical data (Table 1). For the TNBC population, compared with the no-testing and selected testing strategies, the universal testing policy in China resulted in an average life expectancy of 78.57 and 47.38 days gained, respectively, (81.48 and 47.38 days in the USA, respectively). Moreover, the universal testing will result in 0.07 and 0.15 QALYs gained in the Chinese population compared with the no-testing and selected testing scenarios, respectively, with ICERs of 10,592.6 and 10,991.1 USD/QALY, respectively (Fig. 2a). Similarly, for the USA population, the difference in the average QALYs gained between the universal testing and no-testing, as well as that between the universal and selected testing, will be 0.07 and 0.17, respectively, with ICERs of 57,403.0 and 56,518.2 USD/QALY, respectively, (Fig. 2b). The ICERs changing from the selected testing to the universal testing in TNBC (China or the USA) were much lower than the corresponding WTP threshold.

**Table 1:** Average discounted costs, life expectancy and ICERs for base case.

Testing policy	Average discounted costs ($)	Average life expectancy gain			Average quality-adjusted life expectancy gain		
		Years	△Days from reference policy	ICER (cost per year of life expectancy gained; $)	QALYs	△Days from reference policy	ICER (cost per year quality of life gained; $)
TNBC (China)							
No gBRCA test	25,054.75	14.07			8.32		
Selected gBRCA test	25,738.25	14.15	31.26	7980.20	8.39	24.67	10,111.99
Universal gBRCA test	26,634.18	14.28			8.47		
Refer to no test			78.57	7337.09		54.42	10,592.60
Refer to selected test			47.31	6912.14		29.75	10,991.12
TNBC (USA)							
No test	79,956.71	14.06			8.32		
Selected gBRCA test	84,279.86	14.16	34.10	46,267.40	8.39	26.92	58,632.27
Universal gBRCA test	90,069.00	14.29			8.49		
Refer to no test			81.48	45,297.91		64.30	57,403.03
Refer to selected test			47.38	44,600.01		37.39	56,518.17
HER2-negative (China)							
No test	22,554.50	16.78			10.31		
Selected gBRCA test	22,720.68	16.87	30.67	1977.67	10.38	24.85	2440.46
Universal gBRCA test	22,976.54	17.02			10.50		
Refer to no test			87.63	1757.94		71.01	2169.32
Refer to selected test			56.96	1639.62		46.16	2023.31
HER2-negative (USA)							
No test	71,265.75	16.78			10.31		
Selected gBRCA test	72,351.35	16.86	30.47	13,003.36	10.37	24.72	16,029.70
Universal gBRCA test	74,547.72	17.02			10.51		
Refer to no test			89.98	13,313.54		72.98	16,414.19
Refer to selected test			59.51	13,472.37		48.26	16,611.12

*ICER* incremental cost-effectiveness ratio, *QALYs* quality-adjusted life-years.

### Expanding universal testing to all HER2-negative BC patients is cost-effective

**Fig. 3:**
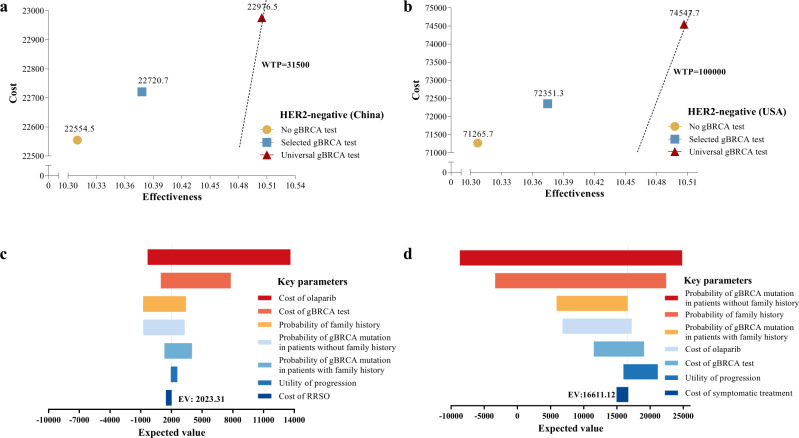
Cost-effectiveness analysis of different gBRCA testing policies in HER2-negative breast cancer patients. **a** Cost-effectiveness ranking (China); **b** Cost-effectiveness ranking (USA); **c** Tornado diagram—net monetary benefits in the sensitivity analysis (China); **d** Tornado diagram—net monetary benefits in the sensitivity analysis (USA).

Considering the tremendous cost-effectiveness of universal testing in all TNBCs, we then expanded the indication of this policy from TNBC to all HER2-negative patients. The universal testing contributed 87.6 and 57.0 days of life expectancy gained in Chinese HER2-negative BC patients compared with patients receiving no testing and the selected testing (Table 1). Similar results were observed in the USA population (90.0 and 59.5 days, respectively). Furthermore, changing from the selected testing to the universal testing could lead to 0.12 and 0.14 QALYs increase, with ICERs of 2023.31 and 16,611.1 USD/QALY in China and the USA, respectively (Fig. 3).

### Sensitivity analysis

**Fig. 4:**
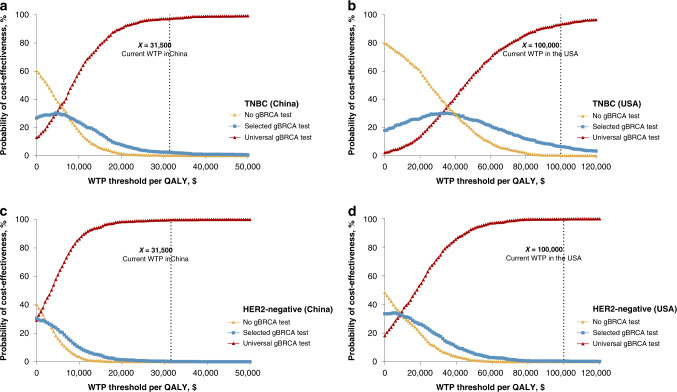
Cost-effectiveness acceptability curves (probabilistic sensitivity analyses). **a** Universal gBRCA testing in all TNBC in China; **b** Universal gBRCA testing in all TNBC in the USA; **c** Universal gBRCA testing in all HER2-negative breast cancer in China; **d** Universal gBRCA testing in all HER2-negative breast cancer in the USA.

Several sensitivity analyses were performed to evaluate the robustness and stability of our results, especially the universal testing policy. The probability of gBRCA mutation, probability of gBRCA mutation in patients without a family history, probability of family history and cost of olaparib contributed significantly to the variation in the results (Figs. 2, 3). Monte Carlo probabilistic sensitivity analysis was stable over a wide range of plausible estimates. The scatters of universal testing still have a seemingly high effectiveness, indicating that the effectiveness of universal testing is robustly high, but the cost is adjustable regarding the olaparib cost (Fig. S1). Figure 4 shows the cost-effectiveness acceptability curve for the probability of the universal gBRCA testing to be cost-effective compared to the other two testing strategies, where there is a high chance for universal gBRCA testing to be cost-effective in all four groups of population (TNBC-CN, TNBC-USA, HER2-negative-CN and HER2-negative-USA). Similarly, the Monte Carlo simulation scatters plot shows that universal testing is cost-effective and under the price of WTP in all populations (Fig. S2).

Once the universal screening policy for HER2-negative BC patients is adopted in China, it will prevent death and recurrence in a substantial number of patients with gBRCA mutations, with 0.3%, 0.9% and 1.5% of the population avoiding death 5, 10 and 20 years after treatment, respectively (Table S5). Comparable results were observed in the USA population (0.2%, 0.7% and 1.3% of the population avoided death 5, 10 and 20 years after treatment, respectively).

## Discussion

### Primary findings

Our analysis demonstrates that gBRCA testing for all patients with HER2-negative breast cancer, including TNBC and HR-positive HER2-negative breast cancer, is cost-effective and has a clinical benefit. To confirm these findings, we performed a stepwise validation of the cost-effectiveness of gBRCA testing and adjuvant olaparib use. Considering the clinical benefits of PARPi and RROs for HER2-negative BC, we assessed the cost-effectiveness of expanding the gBRCA testing to HER2-negative BC patients from TNBC. Surprisingly, moving toward such a universal policy could lead to fewer patients (1.5%/1.3%) suffering from death and more patients (1.7%/1.4%) achieving DF state with an acceptable additional financial burden of 255.9 and 2196.4 USD (ICERs of 2023.3 and 16,611.1 USD/QALY) in China and the USA, respectively. Regarding the WTP in China and the USA being 31,500.0 USD/QALY and 100,000.0 USD /QALY [35], we believe that testing for TNBC and even all HER2-negative BC in China and the USA is cost-effective. Our findings may indicate that the benefits conferred to patients with gBRCA mutations from the adjuvant olaparib and RRO could offset the additional expenses for all such patients to undergo universal testing.

### The gBRCA testing in TNBC/HER2-negative BC patients is potentially cost-effective

The cost-effectiveness of the first-line use of PARPi or the universal testing in the general population of BC is not convincing [36–42]. Tuffaha et al. reported the cost-effectiveness of the gBRCA testing established at the 10% pretest probability threshold [43]; hence expanding the gBRCA test from TNBC to all HER2-negative BC patients is potentially cost-effective, considering the high prevalence (~9.7%) of gBRCA mutations in HER2-negative BC patients.

Elvira et al. found no evidence of cost-effectiveness for gBRCA testing among all newly diagnosed BC followed by cascade testing of relatives [44]. However, Kwon et al. in 2010 revealed the cost-effectiveness in all TNBC patients younger than 50 years old [9, 45]. Although OlympiA did not perform subgroup analysis stratified by age, the entire cohort showed increased benefits from the adjuvant olaparib, with 25% of patients over 50 years old in the trial [9]. This provides sufficient evidence to support the benefits that olaparib confers to these patients [9, 10]. Therefore, the present study did not stratify patients by age, but used a representative cohort to investigate the cost-effectiveness in a clinical setting. Moreover, a recent case-control study reported a high lifetime risk for BC for those with pathogenic variants over 65 years old; despite the survivor bias for these aged patients, Boddicker et al. still provided consistent suggestions that all TNBC patients should receive germline genetic testing [46].

### Universal testing grants survival improvements with acceptable costs

Although significant improvements in the invasive disease-free survival (iDFS) of HER2-negative BC patients receiving adjuvant olaparib have been reported, it could not improve the iDFS of HR-positive HER2-negative BC patients in the subgroup analysis, as reported by the OlympiA study [9]. Comparable results were observed in HR-positive HER2-negative MBC patients [8, 47]. However, this part of patients was still suggested to receive adjuvant olaparib as the recommendation of the updated NCCN guideline. Besides, the benefits of olaparib could be available in metastatic HER2-negative BC, which leads to fewer side effects replacing advanced chemotherapy and increased QoL (quality of life) due to prolonged PFS [8]. In summary, the great benefits of universal testing may be attributable to the use of RRO, adjuvant use of olaparib for high-risk gBRCA-mutant BC and olaparib for BRCA-mutant MBC.

Current guidelines recommend MBC patients receive gBRCA screening for the potential benefits of survival and QoL improvements from PARPi [8, 13]. Though the model we built in the study could not completely reflect the current guidelines of gBRCA screening, especially for those lower than 50 years old who are already recommended to receive gBRCA testing now, the results from our model indeed provide evidence that incorporating PARPi secondary to gBRCA testing for TNBC and HER2-negative BC patients are cost-effective against the WTP in both China and the USA. Herein, it is reasonable to conclude that all TNBC or HER2-negative BC patients shall undergo the gBRCA testing in the early stage, gaining considerable benefits with acceptable costs.

### Implications of universal testing

For breast patients over 45 or 50 years, the current indications for the gBRCA testing based on several clinical characteristics or family history as recommended by different guidelines may have limitations [48]. The selected testing will unavoidably miss numerous gBRCA-mutant BCs with unfavourable prognoses (Table S6) [49]. In our simulation, for family history-based selective testing alone, only 4.30% of TNBC and 3.36% of HER2-negative BC with gBRCA mutations could be detected by the selected testing in China. However, if universal testing is adopted, it is estimated to avoid 6.50% of TNBC and 6.24% of HER2-negative BC with undetected gBRCA mutations. The gBRCA mutations undetected by the selected testing were overestimated compared to previous studies [50]. Nevertheless, Beitsch et al. indicated no statistically significant difference in the probabilities of gBRCA mutation between patients who met the NCCN guidelines and those who did not [50]. Earlier detection of gBRCA mutations could facilitate earlier interventions to promote survival outcomes and avoid additional expenditures due to disease recurrence.

For gBRCA-mutant BC, bilateral mastectomy might be preferable over breast-conserving surgery at the initial surgery consideration [48, 51]. Considering the elevated risk of contralateral BC, bilateral mastectomy could reduce the probability of recurrence and obviate the costs of radiotherapy and secondary surgery [52, 53]. Early detection of gBRCA mutations also provides more adjuvant choices for HER2-negative BC patients. For HER2-negative BC patients, especially those with TNBC, chemotherapy remains the keystone of systemic treatment, while only a few targeted therapies and immunotherapies have been indicated otherwise [13, 54]. Therefore, the universal gBRCA testing could guide clinicians to corresponding treatment and provide alternative targeted therapies to improve survival outcomes [8, 9].

Complex guidelines and malpractice by oncologists may somehow affect the practice of current gBRCA screening policies [55]. Although the screening rates have risen continuously, the estimated screening proportion was still under 50% in patients with a family history of BC or ovarian cancer in 2013 [19, 56]. Hence, the current guideline for the gBRCA testing in BC patients is recommended to be simplified and updated. This may fundamentally render as many gBRCA-mutant TNBC and HR-positive HER2-negative patients as possible to gain benefits. Furthermore, our findings indicate that governments and other healthcare providers need to re-evaluate the accessibility and price setting of gBRCA testing and PARPi. Cheaper gBRCA testing and PARPi as well as easier access to the gBRCA testing are two ways to guarantee the popularisation of universal testing in all HER2-negative patients.

### Comparison of clinical practice in gBRCA testing between China and the USA

The gBRCA screening indications in the updated NCCN guideline (version 1, 2023) of genetic testing for BC patients were consistent with the latest version (2021) of CACA-BC guideline [14, 16]. However, several differences in gBRCA screening indications could be observed. First, all patients with BC under 50 were suggested to receive gBRCA testing by the NCCN guideline, while the CACA-BC guideline recommended that all patients with BC under 45 receive gBRCA testing [14, 16]. This could be explained by considering the age disparity at BC diagnosis between China and the USA (Median age at diagnosis: 50 vs 62 years) [57–59]. Second, the CACA-BC guideline emphasised the importance of age at the diagnosis and genetic counselling, especially history taking on a family history of cancer before gBRCA testing [16]. In the USA, gBRCA testing would be performed before a comprehensive evaluation of patients. Besides, in the latest NCCN guideline, the high-risk HER2-negative BC patients were indicated to receive gBRCA screening once considering the adjuvant usage of PARPi. In addition to the differences between guidelines, the access to genetic testing and healthcare insurance payment also contribute to the difference in clinical practice of gBRCA testing between China and the USA [16, 19, 60].

### Strengths and limitations

For the first time, we indicate that the universal testing for gBRCA-mutant HER2-negative BC patients, together with adjuvant use of PARPi is cost-effective based on the OlympiA trial. This analysis has advantages. First, our model is characterised using a clinical pathway based on NCCN BC or the CACA-BC guidelines [13, 16, 61]. Considering the complexity in the real world, a wide sensitivity range was utilised in the model to accommodate the potential bias; and the simplified and comprehensive screening-diagnosis-treatment strategy modelled in the study might help simplify the guidelines for gBRCA testing and gBRCA-related treatment to increase patient compliance and guideline implementation. Second, multiple scenarios were considered in the model to reflex real-world situations, and the input parameters, both from real-world data and clinical trials, were verified by breast oncologists. In addition, our analysis could provide health economic evidence to the updating NCCN guideline to support the universal gBRCA testing on all HER2-negative BC patients for treatment indications.

Nevertheless, there are limitations to the present study. The study assessed only the cost-effectiveness of different gBRCA testing policies in the USA or China. Nevertheless, for low- and middle-income countries, owing to a lack of real-world data, the results of the present study should be interpreted cautiously. Moreover, we did not run the model and compare cost-effectiveness among different gBRCA screening policies in all BC patients due to limited evidence supporting PARPi in HER2-positive BCs with gBRCA mutations. Besides, we did not model the sensitivity and specificity of gBRCA testing as various literature has reported the gBRCA testing’s positive predictive value being 100% and negative predictive value being 93% or more [62, 63]. To simplify the model, we did not incorporate this variation for modelling the false positives and negatives, but we tried to expand the data range in the sensitivity analysis to compensate for this drawback. Moreover, it is worth noting that the input variables are derived using clinical trial data, which could differ from the effectiveness of treatment in the real world (for instance, the average age of the study population in this analysis was set as 40, same as the cohort of OlympiA trial but lower than the age at diagnosis in the real world). Besides, we only considered conducting mastectomy and bilaterally salpingo-oophorectomy as risk-reducing treatments in the model. Despite that patients with gBRCA PVs are more prone to suffering from endometrial cancer, the incidence is relatively low compared to breast and ovary metastasis, as reported by Kitson et al. [64]. Finally, various screening strategies were not considered in the study (viz., cascade screening or universal genetic screening in community population), as the focus of the study is to investigate the cost-effectiveness of the combination of adjuvant use of olaparib and genetic screening and which genetic screening strategy has the best cost-effectiveness would be a future direction.

## Conclusion

The universal gBRCA testing and adjuvant olaparib are highly cost-effective in TNBC and potentially cost-effective in all HER2-negative BC. Hence the universal testing strategy is recommended for adoption by current guidelines for gBRCA testing. Moreover, owing to the nature of mathematical modelling, the results should be interpreted cautiously.

## Supplementary information

Supplementary figures and tables

Annex I

Supplementary Figure S1

Supplementary Figure S2

CHEERS-checklist

Reproducibility checklist

## Data Availability

The article’s data are available in the manuscript and its online supplementary material.
