# Correction to: All HER2-negative breast cancer patients need gBRCA testing: cost-effectiveness and clinical benefits

**DOI:** 10.1038/s41416-023-02159-4

**Published:** 2023-01-30

**Authors:** Huai-liang Wu, Zi-yin Luo, Zong-lin He, Yue Gong, Miao Mo, Wai-kit Ming, Guang-yu Liu

**Affiliations:** 1 Department of Breast Surgery, Key Laboratory of Breast Cancer in Shanghai, Department of Oncology, Fudan University Shanghai Cancer Center, Shanghai, China; 2 Department of Otorhinolaryngology Head and Neck Surgery, The Sixth Affiliated Hospital of Sun Yat‑sen University, Guangzhou, China; 3 Division of Life Science, The Hong Kong University of Science and Technology, Hong Kong SAR, China; 4 Department of Cancer Prevention, Fudan University Shanghai Cancer Center; Department of Oncology, Shanghai Medical College, Fudan University, Shanghai, China; 5 Department of Infectious Diseases and Public Health, Jockey Club College of Veterinary Medicine and Life Science, City University of Hong Kong, Hong Kong SAR, China

**Keywords:** Health care economics, Breast cancer

Correction to: *British Journal of Cancer* 10.1038/s41416-022-02111-y, published online 23 December 2022

The original version of this article contained a mistake. The first affiliation should be changed to Department of Breast Surgery, Key Laboratory of Breast Cancer in Shanghai, Department of Oncology, Fudan University Shanghai Cancer Center, Shanghai, China. The original article has been corrected.

