# Supplementary material for: All HER2-negative breast cancer patients need gBRCA testing: cost-effectiveness and clinical benefits
Source: Br J Cancer. 2022 Dec 23;128(4):638–46. doi: 10.1038/s41416-022-02111-y (PMC9938252; doi:10.1038/s41416-022-02111-y)
Supplement: Supplementary file 1 — Supplementary figures and tables [file 41416_2022_2111_MOESM1_ESM.docx]

**Supplementary materials**

1. Supplementary Table S1. Summary of key input parameter values and sources in cost-effectiveness analysis of different gBRCA testing policies.
2. Supplementary Table S2. Estimated different proportions of progression without death in different scenarios.
3. Supplementary Table S3. Summarized therapeutic outcomes in different scenarios (different progression/BRCA screening policies).
4. Supplementary Table S4. Medical costs and utility value used in the Markov model in terms of 2021 US dollars.
5. Supplementary Table S5. Clinical outcomes of TNBC and hormonal receptor-positive HER2-negative patients in different scenarios included in the model.
6. Figure S1. Scatterplot of cost-effectiveness analysis. a) TNBC (China); b) TNBC (USA); c) HER2-negative (China); d) HER2-negative (USA).
7. Figure S2. Scatterplot of incremental cost-effectiveness with WTP (Universal testing vs selected testing). a) TNBC (China); b) TNBC (USA); c) HER2-negative (China); d) HER2-negative (USA).

**Supplementary Table S1. Summary of key input parameter values and sources in cost-effectiveness analysis of different gBRCA testing policies.**

| **Variables** | **Probability** | **Range** | **Distribution** | **Reference** |
| --- | --- | --- | --- | --- |
| ***Screening Probabilities of gBRCA mutation*** | | | | |
| **TNBC (China)** |  |  |  |  |
| General population | 0.108 | 0.071-0.161 | Beta | ^1-4^ |
| FH (+) | 0.230 | 0.126-0.230 | Beta | ^3,4^ |
| FH (–) | 0.080 | 0.067-0.111 | Beta | ^3,4^ |
| **HER2-negative (China)** |  |  |  |  |
| General population | 0.096 | 0.065-0.113 | Beta | ^2-5^ |
| FH (+) | 0.200 | 0.126-0.230 | Beta | ^3,4^ |
| FH (–) | 0.075 | 0.067-0.111 | Beta | ^3,4^ |
| **TNBC (USA)** |  |  |  |  |
| General population | 0.112 | 0.084-0.226 | Beta | ^6-10^ |
| FH (+) | 0.252 | 0.227-0.343 | Beta | ^10,11^ |
| FH (–) | 0.080 | 0.066-0.179 | Beta | ^10,11^ |
| **HER2-negative (USA)** |  |  |  |  |
| General population | 0.097 | 0.084-0.226 | Beta | ^6-10^ |
| FH (+) | 0.227 | 0.227-0.343 | Beta | ^10,11^ |
| FH (–) | 0.075 | 0.066-0.179 | Beta | ^10,11^ |
| TNBC (China) patients with family history | 0.187 | 0.100-0.300 | Beta | ^1-4^ |
| HER2-negative (China) patients with family history | 0.168 | 0.100-0.300 | Beta | ^2-5^ |
| TNBC (USA) patients with family history | 0.186 | 0.100-0.300 | Beta | ^6-10^ |
| HER2-negative (USA) patients with family history | 0.145 | 0.100-0.300 | Beta | ^6-10^ |
| TNBC (China) patients with high risk | 0.600 | Fixed | Beta | ^6^ |
| HER2-negative (Chin//a/) patients with high risk | 0.232 | Fixed | Beta | ^2,6^ |
| TNBC (USA) patients with high risk | 0.600 | Fixed | Beta | ^6^ |
| HER2-negative (USA) patients with high risk | 0.201 | Fixed | Beta | ^6,12^ |
| ***Therapeutic outcomes in different scenarios*** | | | | |
| **Undetected BRCA (+) standard treatment (TNBC)** | | | | |
| DFS | 0.931 | ±1% | Beta | ^13,14^ |
| DFS–DFS | 0.931 | ±1% | Beta | ^13,14^ |
| DFS–Recurrence | 0.069 | ±1% | Beta | ^13,14^ |
| DFS–Death | 0 | ±1% | Beta | ^13,14^ |
| Recurrence | 0.069 | ±1% | Beta | ^13,14^, Table S4 and S5 |
| Recurrence–Survival | 0.705 | ±1% | Beta | Table S4 and S5 |
| Recurrence–Death | 0.295 | ±1% | Beta | Table S4 and S5 |
| Death without progression | 0 | ±1% | Beta | ^13,14^ |
| **Undetected BRCA (+) standard treatment (HER2-negative)** | | | | |
| DFS | 0.932 | ±1% | Beta | ^13,14^ |
| DFS–DFS | 0.932 | ±1% | Beta | ^13,14^ |
| DFS–Recurrence | 0.068 | ±1% | Beta | ^13,14^ |
| DFS–Death | 0 | ±1% | Beta | ^13,14^ |
| Recurrence | 0.068 | ±1% | Beta | ^13,14^, Table S4 and S5 |
| Recurrence–Survival | 0.748 | ±1% | Beta | ^15^, Table S4 and S5 |
| Recurrence–Death | 0.252 | ±1% | Beta | ^15^, Table S4 and S5 |
| Death without progression | 0 | ±1% | Beta | ^13,14^ |
| **BRCA (–) standard treatment (TNBC)** | | | | |
| DFS | 0.951 | ±1% | Beta | ^16^ |
| DFS–DFS | 0.951 | ±1% | Beta | ^16^ |
| DFS–Recurrence | 0.048 | ±1% | Beta | ^16^ |
| DFS–Death | 0 | ±1% | Beta | ^16^ |
| Recurrence | 0.048 | ±1% | Beta | ^16^, Table S4 and S5 |
| Recurrence–Survival | 0.698 | ±1% | Beta | ^16^, Table S4 and S5 |
| Recurrence–Death | 0.302 | ±1% | Beta | ^16^, Table S4 and S5 |
| Death without progression | 0 | ±1% | Beta | ^16^ |
| **BRCA (–) standard treatment (HER2-negative)** | | | | |
| DFS | 0.978 | ±1% | Beta | ^17^ |
| DFS–DFS | 0.978 | ±1% | Beta | ^17^ |
| DFS–Recurrence | 0.021 | ±1% | Beta | ^17^ |
| DFS–Death | 0 | ±1% | Beta | Expert opinion |
| Recurrence | 0.021 | ±1% | Beta | ^17^, Table S4 and S5 |
| Recurrence–Survival | 0.739 | ±1% | Beta | ^15^, Table S4 and S5 |
| Recurrence–Death | 0.261 | ±1% | Beta | ^15^, Table S4 and S5 |
| Death without progression | 0 | ±1% | Beta | Expert opinion |
| **BRCA (+) standard treatment (TNBC high risk group)** | | | | |
| DFS | 0.954 | ±1% | Beta | ^13,14^ |
| DFS–DFS | 0.954 | ±1% | Beta | ^13,14^ |
| DFS–Recurrence | 0.044 | ±1% | Beta | ^13,14^ |
| DFS–Death | 0.002 | ±1% | Beta | ^13,14^ |
| Recurrence | 0.044 | ±1% | Beta | ^13,14^, Table S4 and S5 |
| Recurrence–Survival | 0.678 | ±1% | Beta | ^13,14^, Table S4 and S5 |
| Recurrence–Death | 0.322 | ±1% | Beta | ^13,14^, Table S4 and S5 |
| Death without recurrence | 0.002 | ±1% | Beta | ^13,14^ |
| **BRCA (+) standard treatment (HER2-negative high risk group)** | | | | |
| DFS | 0.954 | ±1% | Beta | ^13,14^ |
| DFS–DFS | 0.954 | ±1% | Beta | ^13,14^ |
| DFS–Recurrence | 0.043 | ±1% | Beta | ^13,14^ |
| DFS–Death | 0.002 | ±1% | Beta | ^13,14^ |
| Recurrence | 0.043 | ±1% | Beta | ^13,14^, Table S4 and S5 |
| Recurrence–Survival | 0.726 | ±1% | Beta | ^15^, Table S4 and S5 |
| Recurrence–Death | 0.274 | ±1% | Beta | ^15^, Table S4 and S5 |
| Death without recurrence | 0.002 | ±1% | Beta | ^13,14^ |
| **BRCA (+) standard treatment (TNBC low risk group)** | | | | |
| DFS | 0.966 | ±1% | Beta | ^18^ |
| DFS–DFS | 0.966 | ±1% | Beta | ^18^ |
| DFS–Recurrence | 0.023 | ±1% | Beta | ^18^ |
| DFS–Death | 0.011 | ±1% | Beta | ^18^ |
| Recurrence | 0.023 | ±1% | Beta | ^18^, Table S4 and S5 |
| Recurrence–Survival | 0.678 | ±1% | Beta | ^18^, Table S4 and S5 |
| Recurrence–Death | 0.322 | ±1% | Beta | ^18^, Table S4 and S5 |
| Death without recurrence | 0.011 | ±1% | Beta | ^18^ |
| **BRCA (+) standard treatment (HER2-negative low risk group)** | | | | |
| DFS | 0.970 | ±1% | Beta | ^13,14,19,20^ |
| DFS–DFS | 0.970 | ±1% | Beta | ^13,14,19,20^ |
| DFS–Recurrence | 0.023 | ±1% | Beta | ^19^ |
| DFS–Death | 0.007 | ±1% | Beta | ^19^ |
| Recurrence | 0.023 | ±1% | Beta | ^19^, Table S4 and S5 |
| Recurrence–Survival | 0.726 | ±1% | Beta | ^15^, Table S4 and S5 |
| Recurrence–Death | 0.274 | ±1% | Beta | ^15^, Table S4 and S5 |
| Death without recurrence | 0.007 | ±1% | Beta | ^19^ |

TNBC: Triple negative breast cancer; FH: Family history; DFS: Disease-free survival.

**Supplementary Table S2. Estimated different proportions of recurrence without death in different scenarios.**

| Proportion of recurrence in different scenarios | Second primary malignancies | Recurrence | Distant metastasis | Reference |
| --- | --- | --- | --- | --- |
| Undetected BRCA (+) treatment | 0.521 | 0.083 | 0.396 | ^21-23^ |
| BRCA (–) treatment | 0.180 | 0.225 | 0.595 | ^24^ |
| BRCA (+) treatment | 0.197 | 0.136 | 0.667 | ^13,14^ |

**Supplementary Table S3. Summarized therapeutic outcomes in different scenarios (different recurrence/BRCA screening policies).**

| **Scenarios** | **Overall survival** | **Death** | **Reference** |
| --- | --- | --- | --- |
| **Therapeutic outcomes of TNBC in recurrence** | | | |
| Secondary primary BC | 0.744 | 0.256 | ^25,26^ |
| Local recurrence | 0.866 | 0.134 | ^27^ |
| Distant metastasis | 0.620 | 0.380 | ^28^ |
| **Therapeutic outcomes of HER2-negative patients in recurrence** | | | |
| Secondary primary BC | 0.780 | 0.220 | Estimated |
| Local recurrence | 0.847 | 0.153 | ^27^ |
| Distant metastasis | 0.686 | 0.314 | ^15^ |
| **Therapeutic outcomes in TNBC in different gBRCA testing policies** | | | |
| Undetected BRCA (+) recurrence | 0.705 | 0.295 | Estimated, Table S3 and S4 |
| BRCA (–) recurrence | 0.698 | 0.302 | Estimated, Table S3 and S4 |
| BRCA (+) recurrence | 0.678 | 0.322 | Estimated, Table S3 and S4 |
| **Therapeutic outcomes in HER2-negative patients in different gBRCA testing policies** | | | |
| Undetected BRCA (+) recurrence | 0.748 | 0.252 | Estimated, Table S3 and S4 |
| BRCA (–) recurrence | 0.739 | 0.261 | Estimated, Table S3 and S4 |
| BRCA (+) recurrence | 0.726 | 0.274 | Estimated, Table S3 and S4 |

**Supplementary Table S4. Medical costs and utility value used in the Markov model in terms of 2021 USA dollars.**

|  | **China** | **Range** | **Distribution** | **References** | **USA** | **Range** | **Distribution** | | **References** | |  |
| --- | --- | --- | --- | --- | --- | --- | --- | --- | --- | --- | --- |
| **Cost** |  |  |  |  |  |  |  | |  | |  |
| ***Screening*** |  |  |  |  |  |  |  | |  | |  |
| gBRCA test | 308.6 | ±15% | Gamma | ^29^ | 2496.7 | ±15% | Gamma | | ^23,30^ | |  |
| ***Consultation*** |  |  |  |  |  |  |  | |  | |  |
| Outpatient visit | 7.7 | ±15% | Gamma | ^29^ | 54.2 | ±15% | Gamma | | ^31,32^ | |  |
| Genetic counseling | 23.2 | ±15% | Gamma | ^29^ | 47.1 | ±15% | Gamma | | ^23,33^ | |  |
| Specialist consultation | 85.0 | ±15% | Gamma | ^29^ | 366.9 | ±15% | Gamma | | ^30^ | |  |
| ***Examination*** |  |  |  |  |  |  |  | |  | |  |
| Mammography | 83.09 | ±15% | Gamma | ^29^ | 309.86 | ±15% | Gamma | | ^30^ | |  |
| Ultrasonography | 5.1 | ±15% | Gamma | ^29^ | 141.8 | ±15% | Gamma | | ^34^ | |  |
| CT scan | 52.5 | ±15% | Gamma | ^29^ | 160.2 | ±15% | Gamma | | ^31^ | |  |
| MRI | 78.2 | ±15% | Gamma | ^29^ | 593.0 | ±15% | Gamma | | ^30^ | |  |
| Biopsy | 25.5 | ±15% | Gamma | ^29^ | 291.3 | ±15% | Gamma | | ^34,35^ | |  |
| Pathology with IHC | 308.6 | ±15% | Gamma | ^29^ | 641.7 | ±15% | Gamma | | ^23^ | |  |
| Lab scans and tests (per cycle) | |  |  |  |  | | |  | |  |  |
| Follow-up visit | 180.2 | ±15% | Gamma | ^31^ | 191.9 | ±15% | Gamma | | ^31^ | |  |
| Diagnosed recurrence | 411.2 | ±15% | Gamma | ^31^ | 548.4 | ±15% | Gamma | | ^31^ | |  |
| ***Treatment*** |  |  |  |  |  |  |  | |  | |  |
| Surgery treatment of BC | 3086.4 | ±15% | Gamma | ^29^ | 21431.1 | ±15% | Gamma | | ^23,34^ | |  |
| Salpingo-oophorectomy | 1851.9 | ±15% | Gamma | ^29^ | 7940.7 | ±15% | Gamma | | ^34^ | |  |
| Risk-reducing operation |  |  |  |  |  |  |  | |  | |  |
| RRM | 771.6 | ±15% | Gamma | ^29^ | 13196.3 | ±15% | Gamma | | ^34^ | |  |
| RRSO | 1851.9 | ±15% | Gamma | ^29^ | 7940.7 | ±15% | Gamma | | ^34^ | |  |
| Systemic treatment except PARPi (per cycle) | | |  |  |  |  |  | |  | |  |
| Standard systemic treatment | 2314.6 | ±15% | Gamma | ^36-38^ | 6444.0 | ±15% | Gamma | | ^31,39^ | |  |
| Advanced systemic treatment | 6486.0 | ±15% | Gamma | ^31,36,39^ | 16692.0 | ±15% | Gamma | | ^31,39,40^ | |  |
| Olaparib | 3101.1 | ±15% | Gamma | ^41^ | 15913.7 | ±15% | Gamma | | ^42^ | |  |
| Radiation therapy | 6273.8 | ±15% | Gamma | ^43^ | 8888.3 | ±15% | Gamma | | ^44^ | |  |
| Hospitalization fees (per cycle) |  |  |  |  |  |  |  | |  | |  |
| Before disease recurrence | 532.3 | ±15% | Gamma | ^31^ | 1881.2 | ±15% | Gamma | | ^31,32^ | |  |
| After disease recurrence | 655.0 | ±15% | Gamma | ^31^ | 4864.0 | ±15% | Gamma | | ^31,32^ | |  |
| Nursing (per cycle) |  |  |  |  |  |  |  | |  | |  |
| Before disease recurrence | 112.8 | ±15% | Gamma | ^31^ | 471.3 | ±15% | Gamma | | ^31,32^ | |  |
| After disease recurrence | 157.5 | ±15% | Gamma | ^31^ | 555.3 | ±15% | Gamma | | ^31,32^ | |  |
| Symptomatic treatment | 771.6 | ±15% | Gamma |  | 1749.4 | ±15% | Gamma | | ^23,32^ | |  |
| **Utility** |  |  |  |  |  |  |  | |  | |  |
| Healthy | 0.85 | ±10% | Beta | ^45^ | 0.85 | ±10% | Beta | | ^45^ | |  |
| Recurrence | 0.51 | ±5% | Beta | ^46^ | 0.51 | ±5% | Beta | | ^46^ | |  |
| Death | 0 | Fixed |  | ^45^ | 0 | Fixed |  | | ^45^ | |  |
| PARPi improval in MBC | 0.075 | Fixed | Beta | ^47^ | 0.075 | Fixed | Beta | | ^47^ | |  |
| **Willingness to pay** | 31500.0 | Fixed |  | ^48^ | 100000 | Fixed |  | | ^48^ | |  |
| **Inflation rate** | 0.03 | Fixed |  | ^23^ | 0.03 | Fixed |  | | ^23^ | | |

MRI: Magnetic resonance imaging; IHC: Immunohistochemistry; BC: Breast cancer; RRM: Risk-reducing mastectomy; RRSO: Risk-reducing salpingo-oophorectomy; MBC: Metastatic breast cancer.

**Supplementary Table S5. Clinical outcomes of TNBC and HER2-negative patients (inclusive of HR+HER2- breast cancer and TNBC) in different scenarios included in the model.**

| **Clinical outcomes** | **5 years** | **10 years** | **20 years** |
| --- | --- | --- | --- |
| **TNBC (China)** |  |  |  |
| *Universal BRCA testing* |  |  |  |
| Disease-free survival | 0.782 | 0.611 | 0.374 |
| Recurrence without death | 0.115 | 0.108 | 0.069 |
| Death | 0.104 | 0.281 | 0.557 |
| *Selected BRCA testing* |  |  |  |
| Disease-free survival | 0.775 | 0.600 | 0.361 |
| Recurrence without death | 0.120 | 0.112 | 0.070 |
| Death | 0.106 | 0.288 | 0.569 |
| *No BRCA testing* |  |  |  |
| Disease-free survival | 0.769 | 0.592 | 0.352 |
| Recurrence without death | 0.123 | 0.115 | 0.071 |
| Death | 0.108 | 0.293 | 0.577 |
| **TNBC (USA)** |  |  |  |
| *Universal BRCA testing* |  |  |  |
| Disease-free survival | 0.781 | 0.611 | 0.373 |
| Recurrence without death | 0.115 | 0.109 | 0.069 |
| Death | 0.104 | 0.281 | 0.558 |
| *Selected BRCA testing* |  |  |  |
| Disease-free survival | 0.774 | 0.599 | 0.360 |
| Recurrence without death | 0.120 | 0.113 | 0.070 |
| Death | 0.106 | 0.288 | 0.570 |
| *No BRCA testing* |  |  |  |
| Disease-free survival | 0.770 | 0.593 | 0.353 |
| Recurrence without death | 0.123 | 0.115 | 0.071 |
| Death | 0.107 | 0.292 | 0.576 |
| **HER2-negative (China)** |  |  |  |
| *Universal BRCA testing* |  |  |  |
| Disease-free survival | 0.890 | 0.792 | 0.628 |
| Recurrence without death | 0.063 | 0.070 | 0.059 |
| Death | 0.047 | 0.138 | 0.313 |
| *Selected BRCA testing* |  |  |  |
| Disease-free survival | 0.881 | 0.778 | 0.611 |
| Recurrence without death | 0.070 | 0.075 | 0.061 |
| Death | 0.050 | 0.147 | 0.328 |
| *No BRCA testing* |  |  |  |
| Disease-free survival | 0.876 | 0.771 | 0.603 |
| Recurrence without death | 0.073 | 0.078 | 0.062 |
| Death | 0.051 | 0.151 | 0.335 |
| **HER2-negative (USA)** |  |  |  |
| *Universal BRCA testing* |  |  |  |
| Disease-free survival | 0.890 | 0.792 | 0.628 |
| Recurrence without death | 0.063 | 0.070 | 0.058 |
| Death | 0.047 | 0.138 | 0.313 |
| *Selected BRCA testing* |  |  |  |
| Disease-free survival | 0.882 | 0.780 | 0.614 |
| Recurrence without death | 0.068 | 0.074 | 0.060 |
| Death | 0.049 | 0.145 | 0.326 |
| *No BRCA testing* |  |  |  |
| Disease-free survival | 0.876 | 0.771 | 0.602 |
| Recurrence without death | 0.073 | 0.078 | 0.062 |
| Death | 0.051 | 0.151 | 0.336 |

**Reference**

1. Ji G, Bao L, Yao Q, et al. Germline and tumor BRCA1/2 pathogenic variants in Chinese triple-negative breast carcinomas. *Journal of Cancer Research and Clinical Oncology* 2021: 1-10.

2. Sun J, Meng H, Yao L, et al. Germline mutations in cancer susceptibility genes in a large series of unselected breast cancer patients. *Clinical Cancer Research* 2017; **23**(20): 6113-9.

3. Wang YA, Jian J-W, Hung C-F, et al. Germline breast cancer susceptibility gene mutations and breast cancer outcomes. *BMC cancer* 2018; **18**(1): 1-13.

4. Lang GT, Shi JX, Hu X, et al. The spectrum of BRCA mutations and characteristics of BRCA‐associated breast cancers in China: Screening of 2,991 patients and 1,043 controls by next‐generation sequencing. *International journal of cancer* 2017; **141**(1): 129-42.

5. Zhong X, Dong Z, Dong H, et al. Prevalence and prognostic role of BRCA1/2 variants in unselected Chinese breast cancer patients. *PLoS One* 2016; **11**(6): e0156789.

6. Toss A, Molinaro E, Venturelli M, et al. BRCA detection rate in an Italian cohort of luminal early-onset and triple-negative breast cancer patients without family history: when biology overcomes genealogy. *Cancers* 2020; **12**(5): 1252.

7. Jervis S, Song H, Lee A, et al. A risk prediction algorithm for ovarian cancer incorporating BRCA1, BRCA2, common alleles and other familial effects. *Journal of medical genetics* 2015; **52**(7): 465-75.

8. Couch FJ, Hart SN, Sharma P, et al. Inherited mutations in 17 breast cancer susceptibility genes among a large triple-negative breast cancer cohort unselected for family history of breast cancer. *Journal of clinical oncology* 2015; **33**(4): 304.

9. Shimelis H, LaDuca H, Hu C, et al. Triple-negative breast cancer risk genes identified by multigene hereditary cancer panel testing. *JNCI: Journal of the National Cancer Institute* 2018; **110**(8): 855-62.

10. O’Shaughnessy J, Brezden-Masley C, Cazzaniga M, et al. Prevalence of germline BRCA mutations in HER2-negative metastatic breast cancer: global results from the real-world, observational BREAKOUT study. *Breast Cancer Research* 2020; **22**(1): 1-11.

11. Vig HS, McCarthy AM, Liao K, Demeter MB, Fredericks T, Armstrong K. Age at diagnosis may trump family history in driving BRCA testing in a population of breast cancer patients. *Cancer Epidemiology and Prevention Biomarkers* 2013; **22**(10): 1778-85.

12. DeSantis CE, Ma J, Gaudet MM, et al. Breast cancer statistics, 2019. *CA: a cancer journal for clinicians* 2019; **69**(6): 438-51.

13. Tutt ANJ, Garber JE, Kaufman B, et al. Adjuvant Olaparib for Patients with BRCA1- or BRCA2-Mutated Breast Cancer. *The New England journal of medicine* 2021; **384**(25): 2394-405.

14. Geyer CE, Jr., Garber JE, Gelber RD, et al. Overall survival in the OlympiA phase III trial of adjuvant olaparib in patients with germline pathogenic variants in BRCA1/2 and high-risk, early breast cancer. *Annals of oncology : official journal of the European Society for Medical Oncology* 2022.

15. Vrdoljak E, Marschner N, Zielinski C, et al. Final results of the TANIA randomised phase III trial of bevacizumab after progression on first-line bevacizumab therapy for HER2-negative locally recurrent/metastatic breast cancer. *Annals of oncology* 2016; **27**(11): 2046-52.

16. Copson ER, Maishman TC, Tapper WJ, et al. Germline BRCA mutation and outcome in young-onset breast cancer (POSH): a prospective cohort study. *The lancet oncology* 2018; **19**(2): 169-80.

17. Fehrenbacher L, Cecchini RS, Geyer Jr CE, et al. NSABP B-47/NRG oncology phase III randomized trial comparing adjuvant chemotherapy with or without trastuzumab in high-risk invasive breast cancer negative for HER2 by FISH and with IHC 1+ or 2+. *Journal of Clinical Oncology* 2020; **38**(5): 444.

18. Zhang J, Wang W, Wang J, et al. Survival Outcome and Impact of Chemotherapy in T1 Node-Negative Triple-Negative Breast Cancer: A SEER Database Analysis. *Journal of Oncology* 2020; **2020**.

19. Ruiz-Borrego M, Guerrero-Zotano A, Bermejo B, et al. Phase III evaluating the addition of fulvestrant (F) to anastrozole (A) as adjuvant therapy in postmenopausal women with hormone receptor-positive HER2-negative (HR+/HER2-) early breast cancer (EBC): results from the GEICAM/2006-10 study. *Breast cancer research and treatment* 2019; **177**(1): 115-25.

20. Lambertini M, Ceppi M, Hamy AS, et al. Clinical behavior and outcomes of breast cancer in young women with germline BRCA pathogenic variants. *NPJ breast cancer* 2021; **7**(1): 16.

21. Graeser MK, Engel C, Rhiem K, et al. Contralateral breast cancer risk in BRCA1 and BRCA2 mutation carriers. *Journal of Clinical Oncology* 2009; **27**(35): 5887-92.

22. Kauff ND, Domchek SM, Friebel TM, et al. Risk-reducing salpingo-oophorectomy for the prevention of BRCA1-and BRCA2-associated breast and gynecologic cancer: a multicenter, prospective study. *Journal of Clinical Oncology* 2008; **26**(8): 1331.

23. Kwon JS, Gutierrez-Barrera AM, Young D, et al. Expanding the criteria for BRCA mutation testing in breast cancer survivors. *Journal of clinical oncology* 2010; **28**(27): 4214-20.

24. Li J, Yu K, Pang D, et al. Adjuvant capecitabine with docetaxel and cyclophosphamide plus epirubicin for triple-negative breast cancer (CBCSG010): an open-label, randomized, multicenter, phase III trial. *Journal of Clinical Oncology* 2020; **38**(16): 1774.

25. Ha HI, Lee E-G, Lim J, et al. Second Primary Ovarian Epithelial, Fallopian Tube, and Primary Peritoneal Cancers after Breast Cancer Diagnosis: Korea Central Cancer Registry. *Cancer Research and Treatment: Official Journal of Korean Cancer Association* 2021; **53**(2): 541.

26. Witteveen A, Kwast AB, Sonke GS, IJzerman MJ, Siesling S. Survival after locoregional recurrence or second primary breast cancer: impact of the disease-free interval. *PLoS One* 2015; **10**(4): e0120832.

27. Lee K, Sim SH, Kang EJ, et al. The Role of Chemotherapy in Patients With HER2-Negative Isolated Locoregional Recurrence of Breast Cancer: A Multicenter Retrospective Cohort Study. *Frontiers in oncology* 2021; **11**: 613.

28. Robson M, Tung N, Conte P, et al. OlympiAD final overall survival and tolerability results: Olaparib versus chemotherapy treatment of physician’s choice in patients with a germline BRCA mutation and HER2-negative metastatic breast cancer. *Annals of Oncology* 2019; **30**(4): 558-66.

29. China SHMo. Price Announcement by the Shanghai Health Minister of China in 2017. 2017. <http://wsjkw.sh.gov.cn/ylsfbz/index.html> (accessed Aug. 30 2021).

30. Li Y, Arellano AR, Bare LA, Bender RA, Strom CM, Devlin JJ. A multigene test could cost-effectively help extend life expectancy for women at risk of hereditary breast cancer. *Value in Health* 2017; **20**(4): 547-55.

31. Weng X, Huang X, Li H, et al. First-line treatment with atezolizumab plus nab-paclitaxel for advanced triple-negative breast cancer: a cost-effectiveness analysis. *American journal of clinical oncology* 2020; **43**(5): 340-8.

32. Schwartz KL, Simon MS, Bylsma LC, et al. Clinical and economic burden associated with stage III to IV triple‐negative breast cancer: A SEER‐Medicare historical cohort study in elderly women in the United States. *Cancer* 2018; **124**(10): 2104-14.

33. Manchanda R, Patel S, Antoniou AC, et al. Cost-effectiveness of population based BRCA testing with varying Ashkenazi Jewish ancestry. *Am J Obstet Gynecol* 2017; **217**(5): 578. e1-. e12.

34. Grann VR, Patel PR, Jacobson JS, et al. Comparative effectiveness of screening and prevention strategies among BRCA1/2-affected mutation carriers. *Breast cancer research and treatment* 2011; **125**(3): 837-47.

35. Tosteson AN, Yang Q, Nelson HD, et al. Second opinion strategies in breast pathology: a decision analysis addressing over-treatment, under-treatment, and care costs. *Breast cancer research and treatment* 2018; **167**(1): 195-203.

36. Rui M, Shi F, Shang Y, Meng R, Li H. Economic evaluation of cisplatin plus gemcitabine versus paclitaxel plus gemcitabine for the treatment of first-line advanced metastatic triple-negative breast cancer in China: using Markov model and partitioned survival model. *Advances in therapy* 2020; **37**(9): 3761-74.

37. Wang H, Wang Y, Gong R, Geng Y, Li L. Cost-effectiveness of pertuzumab and trastuzumab as a first-line treatment of HER2-positive metastatic breast cancer in China. *Ann Palliat Med* 2021; **10**(11): 11382-93.

38. Xu Q, Yuanyuan L, Jiejing Z, et al. Cost-effectiveness of paclitaxel, doxorubicin, cyclophosphamide and trastuzumab versus docetaxel, cisplatin and trastuzumab in new adjuvant therapy of breast cancer in china. *Cost Eff Resour Alloc* 2021; **19**(1): 11.

39. Wu B, Ma F. Cost-effectiveness of adding atezolizumab to first-line chemotherapy in patients with advanced triple-negative breast cancer. *Therapeutic Advances in Medical Oncology* 2020; **12**: 1758835920916000.

40. Wheeler SB, Rotter J, Gogate A, et al. Cost-Effectiveness of Pharmacologic Treatment Options for Women With Endocrine-Refractory or Triple-Negative Metastatic Breast Cancer. *Journal of clinical oncology : official journal of the American Society of Clinical Oncology* 2022: JCO2102473.

41. Zhan M, Zheng H, Yang Y, He Z, Xu T, Li Q. Cost-Effectiveness Analysis of Maintenance Olaparib in Patients with Metastatic Pancreatic Cancer and a Germline BRCA1/2 Mutation Based on the POLO Trial. *Cancer Management and Research* 2020; **12**: 12919.

42. Gonzalez R, Havrilesky LJ, Myers ER, et al. Cost-effectiveness analysis comparing “PARP inhibitors-for-all” to the biomarker-directed use of PARP inhibitor maintenance therapy for newly diagnosed advanced stage ovarian cancer. *Gynecologic oncology* 2020; **159**(2): 483-90.

43. Bai Y, Ye M, Cao H, Ma X, Xu Y, Wu B. Economic evaluation of radiotherapy for early breast cancer after breast-conserving surgery in a health resource-limited setting. *Breast cancer research and treatment* 2012; **136**(2): 547-57.

44. Deshmukh AA, Shirvani SM, Lal L, et al. Cost-effectiveness analysis comparing conventional, hypofractionated, and intraoperative radiotherapy for early-stage breast cancer. *JNCI: Journal of the National Cancer Institute* 2017; **109**(11): djx068.

45. Liao M, Jiang Q, Hu H, et al. Cost-effectiveness analysis of utidelone plus capecitabine for metastatic breast cancer in China. *Journal of medical economics* 2019; **22**(6): 584-92.

46. Kim S-H, Jo M-W, Ock M, Lee H-J, Lee J-W. Estimation of health state utilities in breast cancer. *Patient preference and adherence* 2017; **11**: 531.

47. Lloyd A, Nafees B, Narewska J, Dewilde S, Watkins J. Health state utilities for metastatic breast cancer. *British journal of cancer* 2006; **95**(6): 683-90.

48. Bank W. "GDP per capita (current US$)". World Development Indicators. 2021. <https://data.worldbank.org/indicator/NY.GDP.PCAP.CD?most_recent_value_desc=true>.
