# Supplementary material for: All HER2-negative breast cancer patients need gBRCA testing: cost-effectiveness and clinical benefits
Source: Br J Cancer. 2022 Dec 23;128(4):638–46. doi: 10.1038/s41416-022-02111-y (PMC9938252; doi:10.1038/s41416-022-02111-y)
Supplement: Supplementary file 2 — Annex I [file 41416_2022_2111_MOESM2_ESM.docx]

**Annex I. Model building and parameter estimation**

**Model design**

A formal decision tree analytic model based on a transitional Markov chain with clinical data from the Olympia trial and other studies was developed to estimate the costs and benefits of germline BRCA mutation test for a representative cohort of TNBC and hormonal receptor (HR)-positive HER2-negative breast cancer (BC) patients based mainly on the baseline demographic and clinical characteristics of the OlympiA trial ^1,2^. The model consisted of three mutually exclusive health states, namely, disease-free (DF) (the early stage), progression (the advanced stage) ^1^, and death (Figure 1 and Table S1). The DF state indicates the health state where a patient with invasive BC after resectable surgery does not happen events, the duration of which is denoted by disease-free survival (DFS). ^3^ The progression state represents the health state where the patient experiences tumor progression. Death states result from the all-cause mortality of BC patients, so DF patients could also transition to death directly.

Each Markov model cycle was one year, and the time horizon was 20 years. The model starts when 40-year-old women diagnosed with TNBC and HR-positive HER2-negative BC undergo a universal test, a selected test abiding by the current guidelines, or no screening test (Figure 1). A positive gBRCA test plus high-risk stratification will prompt the delivery of olaparib as an adjuvant treatment.

**Model transitions and survival estimates**

TreeAge Pro 2019 (TreeAge, Williamstown, Massachusetts) was used for the Markov model building. Based on several epidemiological investigations of BC patients, the base-case model assumes that the mean age of the affected women is 40 years old ^4-6^. Patients who transitioned between different health states were based on transition probabilities, as shown in Supplementary table S1. Specifically, BRCA1 and BRCA2 mutations were not distinguished. The probability of TNBC women having a gBRCA pathogenic or likely pathogenic variants (P/PVs) was 11.2% for the USA population, ranging from 8.4 and 22.6%^7-11^, and 10.8% for the Chinese population, with a range of 7.1–16.1% ^12-15^. The rate of positive family history among women with TNBC was calculated to be 18.6% in the USA population, and estimated to be 18.7% for the Chinese populations, respectively ^13,16^. For TNBC patients with gBRCA mutation, 34.3% of them had a positive family history positive in the USA population and 23.0% in the Chinese population^17,18^. For HER2-negative patients (inclusive of TNBC and HR-positive HER2-negative BC), gBRCA mutations were found to be present in 9.6% and 9.7% of the Chinese population and USA population, respectively, ranging from 8.4 and 22.6%^7-11^. Positive family history was estimated as 20.0% in the Chinese population and 22.7% in the USA population. A total of 16.8% of HER2-negative patients in China and 14.5% in the USA have a positive family history of gBRCA mutation. The assumptive probability calculation equation is as follows:

$$P_{g}=P_{f}*P_{i}+\left( 1-P_{f} \right)*P_{o},$$

where $P_{g}$ is the probability of gBRCA mutation in the general population. $P_{f}$ is defined as the probability of family history. $P_{i}$ is probability of gBRCA mutation of patients with family history. $P_{o}$is probability of gBRCA mutation of patients without a family history. With the formula above, we then calculate the probability of TNBC patients with a positive family history having gBRCA mutation to be 0.230, and that of those with a negative family history to be 0.08 in China, 0.252 for positive family history and 0.08 for negative family history in USA. Similarly, for HER2-negative patients, the probability of those with a positive family history having gBRCA mutation to be 0.200 and 0.08 for those with a negative family history, 0.227 for positive family history and 0.075 for negative history in USA.

To comply with the clinical situation, in the analytic model, three types of treatment were considered throughout the model: (1) undetected BRCA-positive patients received only standard treatment; (2) BRCA-negative patients received only standard treatment; and (3) identified BRCA-positive patients received standard treatment, with PARPi (olaparib 600 mg/day orally) for high-risk patients and RRT for certain proportions. We assumed that all the affected women would undergo standard treatment following the NCCN and the Chinese Anti-Cancer Association Committee of Breast Cancer Society (CACA-BC) guidelines^19,20^. Standard treatment is defined as local treatment plus systemic treatment following the current guideline of breast cancer treatment after the original diagnosis. Besides, to estimate the optimized benefit of gBRCA mutation testing, we assumed that a great portion of all confirmed mutation carriers would choose to undergo risk-reducing treatments (RRT) including risk-reducing mastectomy (RRM) and risk-reducing bilaterally salpingo-oophorectomy (RRBSO) ^21-25^. According to the clinical data from OlympiA, we estimated 40% and 60% of confirmed mutation carriers will also undergo RRM and RRBSO respectively ^1^. In this sense, a portion of the women will remain at risk for subsequent contralateral breast cancer, and ovarian cancer ^26^.

Transition probabilities were calculated for each cycle for the occurrence of events. The one-year transition probability was derived from survival rates and mortality rates using the formula below:

$$probability= 1-e^{(-rt)}$$

where r is rate and t is the time interval^27-29^.

For undetected BRCA-positive patients, we assume that the 1-year DFS rate is 0.931 in TNBC patients by a series of calculations, which used the weighted mean of invasive disease-free survival, and 0.932 in HER2-negative patients considering the high probability of ipsilateral breast cancer and ovarian cancer (Supplementary table S1). Clinical outcomes of patients in the arm of BRCA-positive standard treatment are referred to the olaparib arm in the OlympiA trial^1,2^. In the arm of BRCA-negative patients, the annual DFS probability of TNBC patients after standard treatment is estimated to be 0.951, according to the POSH trial ^30^ and 0.978 for HER2-negative patients retrieved from the NSABP trial^31^. For BRCA-mutant patients, the survival outcomes of high-risk TNBC and HR-positive HER2-negative BC were available from OlympiA^1^, while low-risk TNBC was estimated to be 0.966 for 1-year DFS from a retrospective analysis of the Surveillance, Epidemiology, and End Results (SEER) database ^32^. However, due to a lack of real-world data, we had to estimate the annual DFS of HER2-negative BC to be 0.970 based on clinical opinions, which is assumed by the fact that the prognosis of HER2-negative patients is a bit greater than TNBC patients. Furthermore, we estimate the probability of death without any events as 0. As for the probability of survival after progression, we have found that it is about 0.739 in HER2-negative patients based on the TANIA trial^33^, whereas we assume 0.698 in TNBC patients for the reason that TNBC patients have a worse prognosis ^34^.

Once progression is experienced in BRCA-mutant BC, olaparib will be administered and maintained for one year ^35^. However, if these BRCA-mutant BC have already received adjuvant olaparib, they would not be given again upon progression, considering the drug tolerance. The progression probability was calculated and merged from 3 possible progression states (locoregional recurrence, secondary primary malignancies, and distant metastasis) reported in the literature (Table S3 and S4). All patients are expected to receive the best supportive treatment to curb progression until death. The annual mortality rate per cycle was calculated from the 5-year mortality rate reported by the OlympiA and relevant clinical trials ^1^, and all rates were converted into annual probabilities using the rate to probability formula (1 – e ^− rate × time^) where relevant. Moreover, we assume that once patients experience progression, different management strategies are considered from a clinic perspective as follows.

For secondary primary malignancies, we assume half of these patients would have primary ovarian cancer and the other half have contralateral breast cancer. Hence, for the costs of secondary primary malignancies, 50% of the fees were for ovarian cancer (standard salpingo-oophorectomy plus systemic treatment), and 50% of the fees were for contralateral breast cancer (mastectomy plus systemic treatment). For locoregional recurrence, an estimated half of patients with unresectable recurrence would receive radiotherapy with standard systemic treatment, while half of the patients with resectable recurrence would undergo standard breast cancer surgery with standard systemic treatment. For distant metastasis, patients would be considered advanced chemotherapy ^35,36^. So here we assumed all patients with distant metastasis will undergo chemotherapy, and undergo regular outpatients and inpatient visit. We assumed that all patients received their assigned treatment in the DFS state until disease progression or death without events.

**Definition of variables included in the cost-effectiveness analysis.**

TNBC is defined as the negativity of estrogen-receptor, progesterone-receptor, and HER2 status.

gBRCA mutation refers to the mutation of either germline BRCA1 or BRCA2 gene.

DFS (Disease-free survival) is defined as the time from completed remission (resectable surgery) until the first event or date of last follow-up ^3^. PFS (Progression-free survival) is defined as the time elapsed between treatment initiation and tumor progression or death from any cause, with censoring of patients who are lost to follow-up ^37^.

High-risk TNBC is defined as the non-PCR (pathological complete response) in neoadjuvant patients or patients with the axillary node-positive disease or an invasive primary tumor measuring at least 2 cm on pathological analysis^1,2^. It covers approximately 60% of all TNBC patients ^38,39^. High-risk HR-positive HER2-negative patients are required to have at least four pathologically confirmed positive lymph nodes or have non-PCR with a CPS+EG score of 3 or higher^1,2^. It is estimated to account for 14% of all HR-positive HER2-negative breast cancer patients ^40^.

**Cost estimates**

The costs associated with standard breast cancer surgery include the fees for biopsy, mammography, MRI, surgery, pathology with IHC (immunohistochemistry), specialist consultation, and follow-up visit. The costs associated with progression include fees for standard local and systemic treatment, symptomatic treatment, nursing, imaging examination, laboratory investigation, hospitalization, and outpatient visits.

All costs in China were extracted from the price announcement by Shanghai Health Minister of China in 2017 ^41^, unless specified, as shown in Supplementary Table S4. For costs in USA, the majority of the parameters were sourced from previously published literature^1^. The base costs were inflated to reflect 2021 USD using the Consumer Price Index inflation calculator from the World Bank data ^42^, and the costs and utilities were discounted at a 3% annual rate to account for inflation ^43^.

Specifically, in the model, all patients will have a one-time expenditure for undergoing pre-operative examination (Surgery specialist consultation, biopsy, MRI, laboratory tests, ultrasound, CT scan, and mammography), standard treatment (Standard breast surgery, radiation therapy, chemotherapy), symptomatic treatment, post-operative immunohistochemistry pathology with and follow-up (outpatient visit, routine laboratory tests), and management fees (nursing, and hospitalization), as summarized in Supplementary Table S4. Moreover, they would have yearly recurring medical expenditure for laboratory testing, ultrasound examination, mammography, and two outpatient visits. Specifically, the fees for laboratory testing, hospitalization as well as nursing before and after progression are different, and all the values were retrieved from literature ^44,45^. Later, upon progression, all patients will have yearly recurring laboratory testing (post-progression), mammography, ultrasound, 2-time outpatient visits, symptomatic treatment, and hospitalization (after progression), as well as fees for nursing (post-progression), until death.

To be more specific, for the 3 types of patients in the model (undetected gBRCA positive, gBRCA positive, and gBRCA negative), we have three screening strategies on gBRCA mutation assessed in the model (no testing, selected testing, and universal testing), and they have different additional medical expenditure as follows:

1) No testing group: As they are the patients having no family history test and universal gBRCA screening, they would not have additional medical fees upon primary treatment. But upon progression, patients without prior genetic testing will undergo gBRCA test, and genetic counselling, together with post-progression chemotherapy. For those with positive gBRCA mutation, once they belong to high-risk HER2-negative breast cancer or TNBC, they would also receive one-year-long olaparib treatment; for those without gBRCA mutation, they have no additional treatment.

2) Selected testing group: Similarly, for patients with family history, they would undergo genetic counselling at the very beginning upon first surgery. Moreover, only for those with positive family history whose immediate relatives have gBRCA mutations, would they undergo gBRCA test.

Hence there would be a group of patients without family history who with undetected positive gBRCA mutations, who would undergo gBRCA test upon progression, and receive olaparib only when progression. Later, once detected for positive gBRCA mutations, they would be recommended to undergo risk-reducing treatment (risk-reducing mastectomy and risk-reducing salpingo-oophorectomy), but due to various reasons (socio-economic, religio-cultural, or aesthetic), only a fraction of patients (estimated 40% for RRM and 60% for RRBSO) would undergo risk-reducing treatment, hence a discounted fee for RRT were considered and accounted for these patients. Moreover, only high-risk HER2-negative breast cancer and TNBC would receive adjuvant olaparib at the very beginning, and if not, then they would only receive the 12-month-long olaparib treatment upon progression. Moreover, for those high-risk patients completed adjuvant olaparib treatment but still progress, it is expected that they are resistant to PARPi, and olaparib would not be given, and we assumed they received other types of adjuvant therapy instead upon progression.

3) Universal testing group: Patients in this group will receive gBRCA testing when receiving first standard treatment. Similar to those in family history group, low-risk patients will receive RRT together with primary standard surgery, and only receive 12-month-long olaparib treatment upon progression, while high-risk patients will receive 12-month-long olaparib treatment, RRT as well as standard treatment before progression, and they would receive other adjuvant therapy upon progression.

Patients who survive upon progression will spend money on outpatient visits, follow-up visits, and lab investigations yearly in each cycle. Costs for the management of adverse events were based on previously published studies^1^, as shown in Table S4. All the costs were transformed into annual costs using the median costs spent yearly by breast cancer patients, as suggested by expert opinion and relevant studies ^46,47^. For these calculations, the CNY exchange rate against the USD is 6.48, referring to the daily exchange rate on July 21, 2021.

**Reference**

1. Tutt AN, Garber JE, Kaufman B, et al. Adjuvant Olaparib for Patients with BRCA1-or BRCA2-Mutated Breast Cancer. *New England Journal of Medicine* 2021.

2. Geyer CE, Jr., Garber JE, Gelber RD, et al. Overall survival in the OlympiA phase III trial of adjuvant olaparib in patients with germline pathogenic variants in BRCA1/2 and high-risk, early breast cancer. *Annals of oncology : official journal of the European Society for Medical Oncology* 2022.

3. Creutzig U, Zimmermann M, Ritter J, et al. Definition of a standard-risk group in children with AML. *Br J Haematol* 1999; **104**(3): 630-9.

4. Chen S, Parmigiani G. Meta-analysis of BRCA1 and BRCA2 penetrance. *Journal of clinical oncology: official journal of the American Society of Clinical Oncology* 2007; **25**(11): 1329.

5. Collins IM, Milne RL, Weideman PC, et al. Preventing breast and ovarian cancers in high‐risk BRCA1 and BRCA2 mutation carriers. *Medical Journal of Australia* 2013; **199**(10): 680-3.

6. Rhiem K, Engel C, Graeser M, et al. The risk of contralateral breast cancer in patients from BRCA1/2 negative high risk families as compared to patients from BRCA1 or BRCA2 positive families: a retrospective cohort study. *Breast cancer research* 2012; **14**(6): 1-8.

7. Toss A, Molinaro E, Venturelli M, et al. BRCA detection rate in an Italian cohort of luminal early-onset and triple-negative breast cancer patients without family history: when biology overcomes genealogy. *Cancers* 2020; **12**(5): 1252.

8. Shimelis H, LaDuca H, Hu C, et al. Triple-negative breast cancer risk genes identified by multigene hereditary cancer panel testing. *JNCI: Journal of the National Cancer Institute* 2018; **110**(8): 855-62.

9. Couch FJ, Hart SN, Sharma P, et al. Inherited mutations in 17 breast cancer susceptibility genes among a large triple-negative breast cancer cohort unselected for family history of breast cancer. *Journal of clinical oncology* 2015; **33**(4): 304.

10. Jervis S, Song H, Lee A, et al. A risk prediction algorithm for ovarian cancer incorporating BRCA1, BRCA2, common alleles and other familial effects. *Journal of medical genetics* 2015; **52**(7): 465-75.

11. O’Shaughnessy J, Brezden-Masley C, Cazzaniga M, et al. Prevalence of germline BRCA mutations in HER2-negative metastatic breast cancer: global results from the real-world, observational BREAKOUT study. *Breast Cancer Research* 2020; **22**(1): 1-11.

12. Ji G, Bao L, Yao Q, et al. Germline and Tumor BRCA1/2 Pathogenic Variants in Chinese Triple-negative Breast Carcinomas. 2021.

13. Lang GT, Shi JX, Hu X, et al. The spectrum of BRCA mutations and characteristics of BRCA‐associated breast cancers in China: Screening of 2,991 patients and 1,043 controls by next‐generation sequencing. *International journal of cancer* 2017; **141**(1): 129-42.

14. Sun J, Meng H, Yao L, et al. Germline mutations in cancer susceptibility genes in a large series of unselected breast cancer patients. *Clinical Cancer Research* 2017; **23**(20): 6113-9.

15. Wang YA, Jian J-W, Hung C-F, et al. Germline breast cancer susceptibility gene mutations and breast cancer outcomes. *BMC cancer* 2018; **18**(1): 1-13.

16. Mori H, Kubo M, Kai M, et al. BRCAness combined with a family history of cancer is associated with a poor prognosis for breast cancer patients with a high risk of BRCA mutations. *Clinical breast cancer* 2018; **18**(5): e1217-e27.

17. Vig HS, McCarthy AM, Liao K, Demeter MB, Fredericks T, Armstrong K. Age at diagnosis may trump family history in driving BRCA testing in a population of breast cancer patients. *Cancer Epidemiol Biomarkers Prev* 2013; **22**(10): 1778-85.

18. O'Shaughnessy J, Brezden-Masley C, Cazzaniga M, et al. Prevalence of germline BRCA mutations in HER2-negative metastatic breast cancer: global results from the real-world, observational BREAKOUT study. *Breast cancer research : BCR* 2020; **22**(1): 114.

19. Chinese Anti-cancer Association. Guidelines for the diagnosis and treatment of breast cancer by Chinese Anti-cancer Association (2019 edition). *China Oncology* 2019; **29**(8): 609-80.

20. Daly MB, Pal T, Berry MP, et al. Genetic/Familial High-Risk Assessment: Breast, Ovarian, and Pancreatic, Version 1.2023, NCCN Clinical Practice Guidelines in Oncology. 2022. <https://www.nccn.org/guidelines/guidelines-detail?category=2&id=1503> (accessed Nov 4 2022).

21. Foster C, Watson M, Eeles R, et al. Predictive genetic testing for BRCA1/2 in a UK clinical cohort: three-year follow-up. *British journal of cancer* 2007; **96**(5): 718-24.

22. Bradbury AR, Ibe CN, Dignam JJ, et al. Uptake and timing of bilateral prophylactic salpingo-oophorectomy among BRCA1 and BRCA2 mutation carriers. *Genetics in Medicine* 2008; **10**(3): 161-6.

23. Botkin JR, Smith KR, Croyle RT, et al. Genetic testing for a BRCA1 mutation: prophylactic surgery and screening behavior in women 2 years post testing. *American journal of medical genetics Part A* 2003; **118**(3): 201-9.

24. Friebel TM, Domchek SM, Neuhausen SL, et al. Bilateral prophylactic oophorectomy and bilateral prophylactic mastectomy in a prospective cohort of unaffected BRCA1 and BRCA2 mutation carriers. *Clinical breast cancer* 2007; **7**(11): 875-82.

25. Meijers-Heijboer E, Verhoog L, Brekelmans C, et al. Presymptomatic DNA testing and prophylactic surgery in families with a BRCA1 or BRCA2 mutation. *The Lancet* 2000; **355**(9220): 2015-20.

26. Graeser MK, Engel C, Rhiem K, et al. Contralateral breast cancer risk in BRCA1 and BRCA2 mutation carriers. *Journal of Clinical Oncology* 2009; **27**(35): 5887-92.

27. Neumann PJ, Sanders GD, Russell LB, Siegel JE, Ganiats TG. Cost-effectiveness in health and medicine: Oxford University Press; 2016.

28. Miller DK, Homan SM. Determining transition probabilities: confusion and suggestions. *Medical Decision Making* 1994; **14**(1): 52-8.

29. Jones E, Epstein D, García-Mochón L. A procedure for deriving formulas to convert transition rates to probabilities for multistate Markov models. *Medical Decision Making* 2017; **37**(7): 779-89.

30. Copson ER, Maishman TC, Tapper WJ, et al. Germline BRCA mutation and outcome in young-onset breast cancer (POSH): a prospective cohort study. *The Lancet Oncology* 2018; **19**(2): 169-80.

31. Fehrenbacher L, Cecchini RS, Geyer CE, Jr., et al. NSABP B-47/NRG Oncology Phase III Randomized Trial Comparing Adjuvant Chemotherapy With or Without Trastuzumab in High-Risk Invasive Breast Cancer Negative for HER2 by FISH and With IHC 1+ or 2. *Journal of clinical oncology : official journal of the American Society of Clinical Oncology* 2020; **38**(5): 444-53.

32. Zhang J, Wang W, Wang J, et al. Survival Outcome and Impact of Chemotherapy in T1 Node-Negative Triple-Negative Breast Cancer: A SEER Database Analysis. *J Oncol* 2020; **2020**: 8880727.

33. Vrdoljak E, Marschner N, Zielinski C, et al. Final results of the TANIA randomised phase III trial of bevacizumab after progression on first-line bevacizumab therapy for HER2-negative locally recurrent/metastatic breast cancer. *Annals of oncology : official journal of the European Society for Medical Oncology* 2016; **27**(11): 2046-52.

34. Dent R, Trudeau M, Pritchard KI, et al. Triple-negative breast cancer: clinical features and patterns of recurrence. *Clinical cancer research : an official journal of the American Association for Cancer Research* 2007; **13**(15 Pt 1): 4429-34.

35. Gradishar WJ, Moran MS, Abraham J, et al. NCCN Guidelines® Insights: Breast Cancer, Version 4.2022. 2022. <https://www.nccn.org/professionals/physician_gls/pdf/breast.pdf> (accessed 4 Nov 2022.

36. FDA Approves KEYTRUDA® (pembrolizumab) for Treatment of Patients With High-Risk Early-Stage Triple-Negative Breast Cancer in Combination With Chemotherapy as Neoadjuvant Treatment, Then Continued as Single Agent as Adjuvant Treatment After Surgery. 2021. <https://www.merck.com/news/fda-approves-keytruda-pembrolizumab-for-treatment-of-patients-with-high-risk-early-stage-triple-negative-breast-cancer-in-combination-with-chemotherapy-as-neoadjuvant-treatment-then-continued/> (accessed Seq 18 2021).

37. Saad ED, Katz A. Progression-free survival and time to progression as primary end points in advanced breast cancer: often used, sometimes loosely defined. *Annals of oncology : official journal of the European Society for Medical Oncology* 2009; **20**(3): 460-4.

38. Leon-Ferre RA, Polley MY, Liu H, et al. Impact of histopathology, tumor-infiltrating lymphocytes, and adjuvant chemotherapy on prognosis of triple-negative breast cancer. *Breast cancer research and treatment* 2018; **167**(1): 89-99.

39. Denkert C, von Minckwitz G, Darb-Esfahani S, et al. Tumour-infiltrating lymphocytes and prognosis in different subtypes of breast cancer: a pooled analysis of 3771 patients treated with neoadjuvant therapy. *The Lancet Oncology* 2018; **19**(1): 40-50.

40. Pohl-Rescigno E, Hauke J, Loibl S, et al. Association of Germline Variant Status With Therapy Response in High-risk Early-Stage Breast Cancer: A Secondary Analysis of the GeparOcto Randomized Clinical Trial. *JAMA Oncol* 2020; **6**(5): 744-8.

41. Shanghai Municipal Health Commission. Medical service items and prices in the medical institutions from Shanghai (September 2017). 2017. <http://wsjkw.sh.gov.cn/ylsfbz/index.html> (accessed 08/02 2021).

42. The World Bank. World Bank Open Data. 2021. <https://data.worldbank.org/> (accessed 08/02 2021).

43. Bank TW. World Bank Open Data. 2021. <https://data.worldbank.org/>.

44. Weng X, Huang X, Li H, et al. First-line treatment with atezolizumab plus nab-paclitaxel for advanced triple-negative breast cancer: a cost-effectiveness analysis. *American journal of clinical oncology* 2020; **43**(5): 340-8.

45. Schwartz KL, Simon MS, Bylsma LC, et al. Clinical and economic burden associated with stage III to IV triple‐negative breast cancer: A SEER‐Medicare historical cohort study in elderly women in the United States. *Cancer* 2018; **124**(10): 2104-14.

46. Kwon JS, Gutierrez-Barrera AM, Young D, et al. Expanding the criteria for BRCA mutation testing in breast cancer survivors. *Journal of clinical oncology* 2010; **28**(27): 4214-20.

47. Li Y, Arellano AR, Bare LA, Bender RA, Strom CM, Devlin JJ. A multigene test could cost-effectively help extend life expectancy for women at risk of hereditary breast cancer. *Value in Health* 2017; **20**(4): 547-55.
