# Supplementary figures and images for: All HER2-negative breast cancer patients need gBRCA testing: cost-effectiveness and clinical benefits
Source: Br J Cancer. 2022 Dec 23;128(4):638–46. doi: 10.1038/s41416-022-02111-y (PMC9938252; doi:10.1038/s41416-022-02111-y)

Supplementary Figure S1

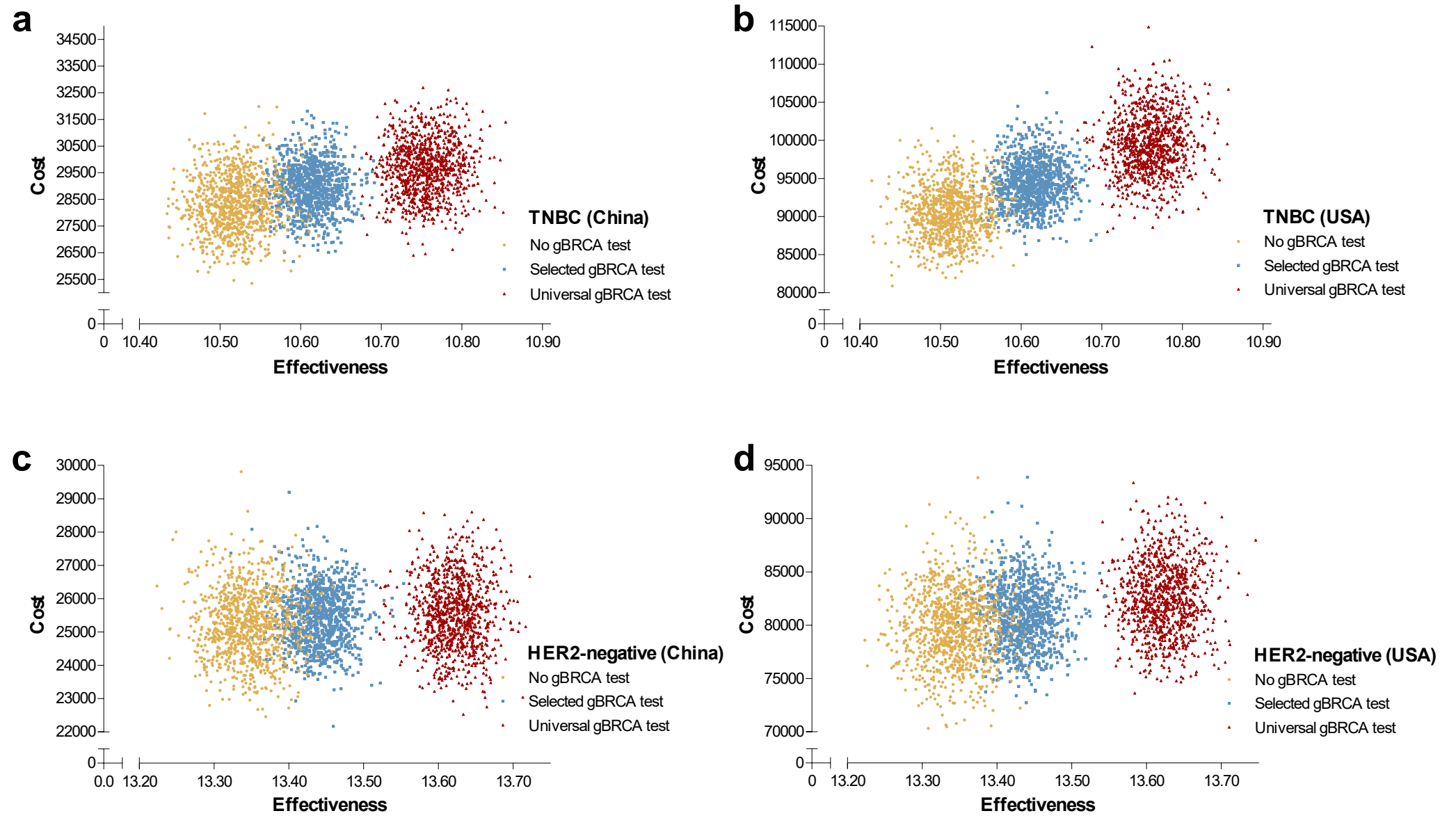

Supplement: Supplementary file 3 — Supplementary Figure S1 [file 41416_2022_2111_MOESM3_ESM.pdf]

Supplementary Figure S2

a

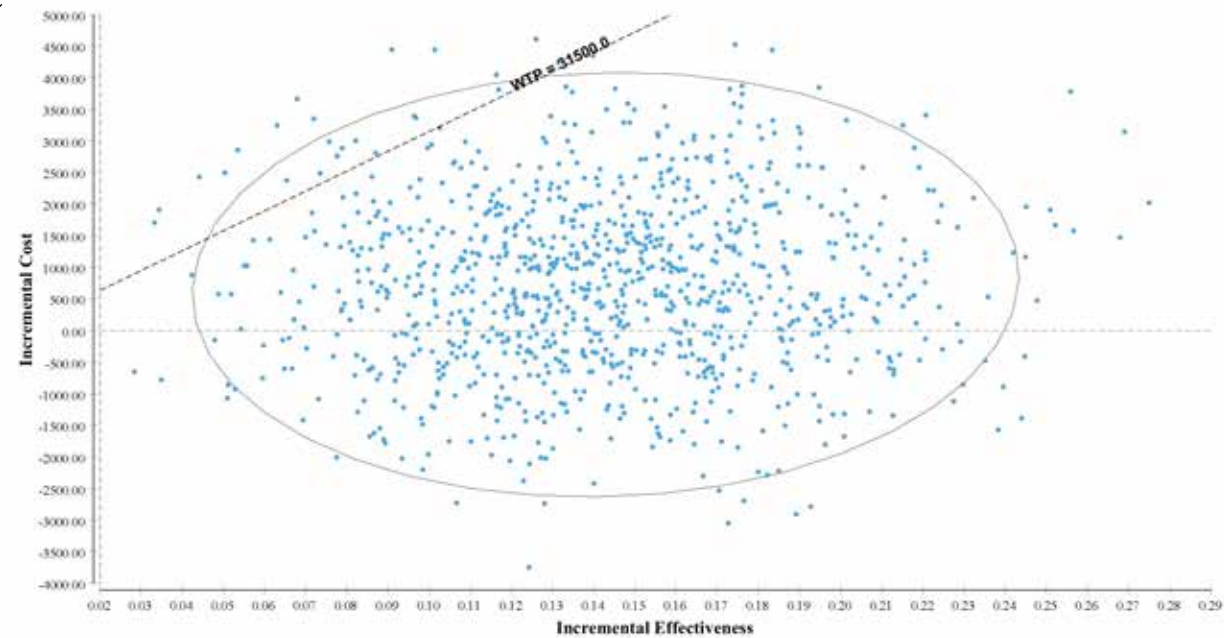

b

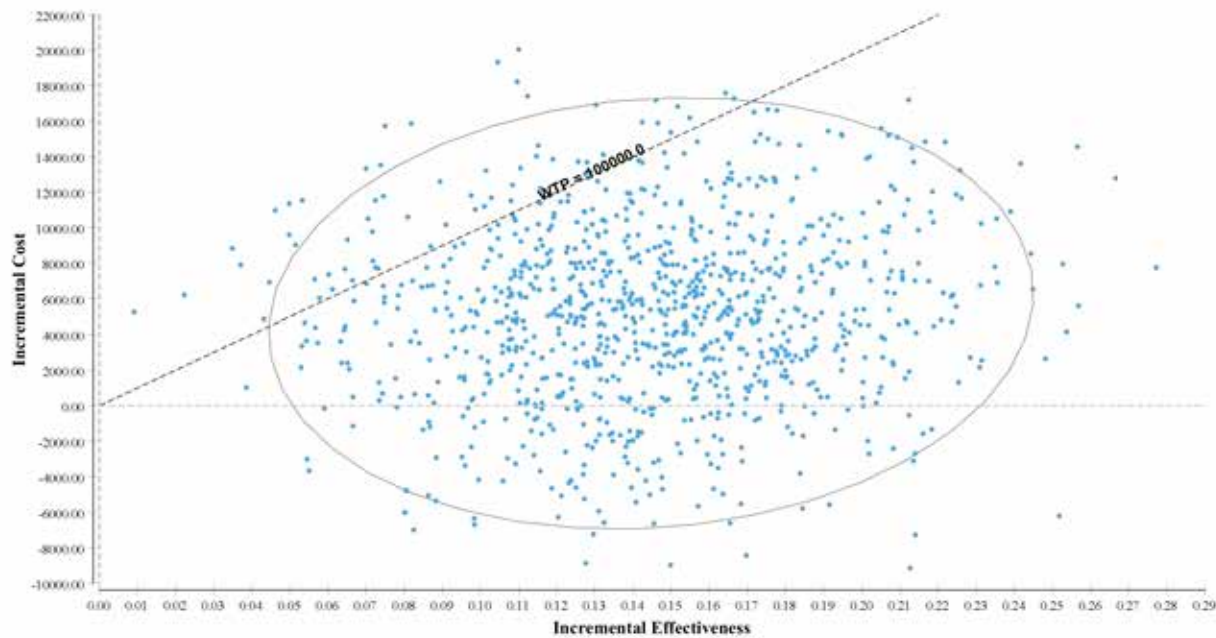

c

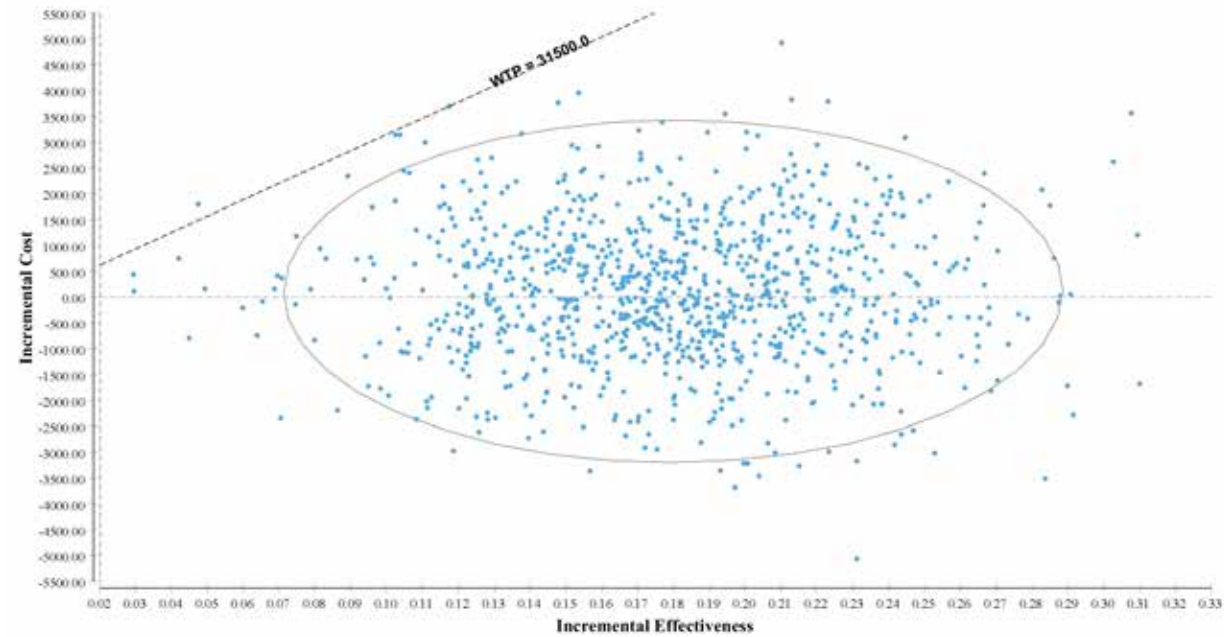

d

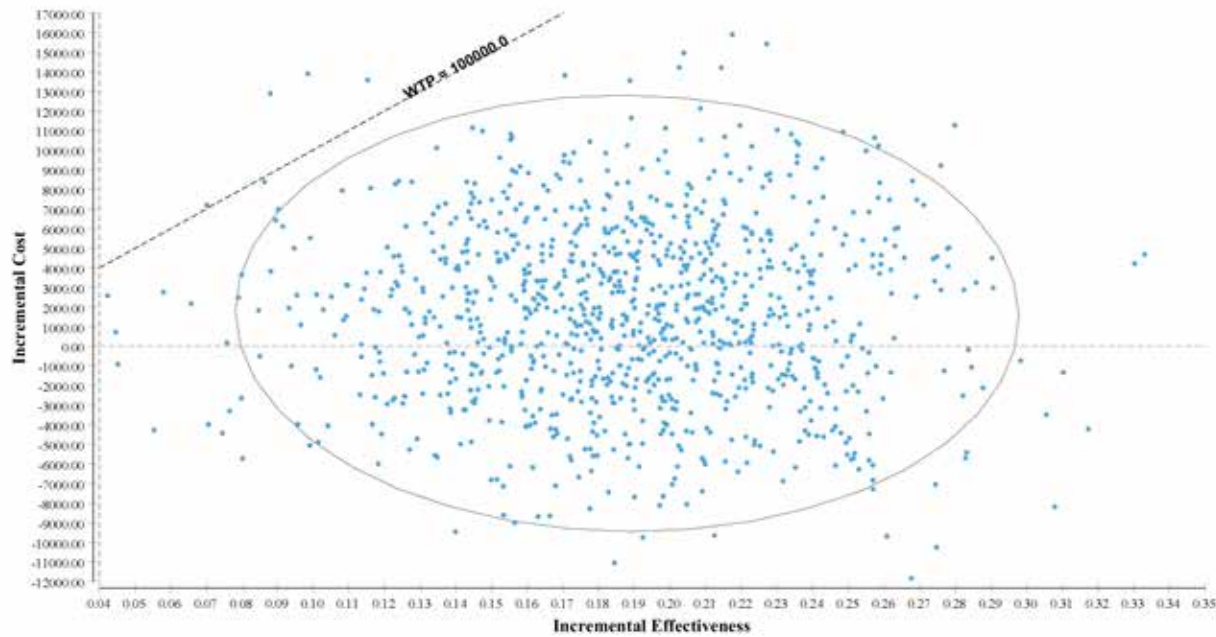

Supplement: Supplementary file 4 — Supplementary Figure S2 [file 41416_2022_2111_MOESM4_ESM.pdf]
